# Distinct profiles of proliferating CD8+/TCF1+ T cells and CD163+/PD-L1+ macrophages predict risk of relapse differently among treatment-naïve breast cancer subtypes

**DOI:** 10.1007/s00262-024-03630-8

**Published:** 2024-02-13

**Authors:** Konstantinos Ntostoglou, Sofia D. P. Theodorou, Tanja Proctor, Ilias P. Nikas, Sinclair Awounvo, Athanasia Sepsa, Vassilis Georgoulias, Han Suk Ryu, Ioannis S. Pateras, Christos Kittas

**Affiliations:** 1 Department of Histopathology, Biomedicine Group of Health Company, 15626 Athens, Greece; 2 Medical School, National and Kapodistrian University of Athens, 11527 Goudi, Athens, Greece; 3 Institute of Medical Biometry, University of Heidelberg, 69120 Heidelberg, Germany; 4 Medical School, University of Cyprus, 2029 Nicosia, Cyprus; 5 Department of Anatomic Pathology, Metropolitan Hospital, 9 Ethnarchou Makariou & 1 E. Venizelou Street, Neo Faliro, 18547 Piraeus, Greece; 6 Hellenic Research Oncology Group, 11474 Athens, Greece; 7 Department of Pathology, College of Medicine, Seoul National University Hospital, 03080 Seoul, Republic of Korea; 8 2nd Department of Pathology, Medical School, “Attikon” University Hospital, National and Kapodistrian University of Athens, 124 62 Athens, Greece

**Keywords:** Tumor-infiltrating lymphocytes, T cell state, Tumor-associated macrophages, Prognosis, Luminal breast cancer, Triple-negative breast cancer

## Abstract

Immunophenotypic analysis of breast cancer microenvironment is gaining attraction as a clinical tool improving breast cancer patient stratification. The aim of this study is to evaluate proliferating CD8 + including CD8 + TCF1 + Τ cells along with PD-L1 expressing tissue-associated macrophages among different breast cancer subtypes. A well-characterized cohort of 791 treatment-naïve breast cancer patients was included. The analysis demonstrated a distinct expression pattern among breast cancer subtypes characterized by increased CD8 + , CD163 + and CD163 + PD-L1 + cells along with high PD-L1 status and decreased fraction of CD8 + Ki67 + T cells in triple negative (TNBC) and HER2 + compared to luminal tumors. Kaplan–Meier and Cox univariate survival analysis revealed that breast cancer patients with high CD8 + , CD8 + Ki67 + , CD8 + TCF1 + cells, PD-L1 score and CD163 + PD-L1 + cells are likely to have a prolonged relapse free survival, while patients with high CD163 + cells have a worse prognosis. A differential impact of high CD8 + , CD8 + Ki67 + , CD8 + TCF1 + T cells, CD163 + PD-L1 + macrophages and PD-L1 status on prognosis was identified among the various breast cancer subtypes since only TNBC patients experience an improved prognosis compared to patients with luminal A tumors. Conversely, high infiltration by CD163 + cells is associated with worse prognosis only in patients with luminal A but not in TNBC tumors. Multivariate Cox regression analysis in TNBC patients revealed that increased CD8 + [hazard ratio (HR) = 0.542; 95% confidence interval (CI) 0.309–0.950; *p* = 0.032), CD8 + TCF1 + (HR = 0.280; 95% CI 0.101–0.779; *p* = 0.015), CD163 + PD-L1 + (HR: 0.312; 95% CI 0.112–0.870; *p* = 0.026) cells along with PD-L1 status employing two different scoring methods (HR: 0.362; 95% CI 0.162–0.812; *p* = 0.014 and HR: 0.395; 95% CI 0.176–0.884; *p* = 0.024) were independently linked with a lower relapse rate. Multivariate analysis in Luminal type A patients revealed that increased CD163 + was independently associated with a higher relapse rate (HR = 2.360; 95% CI 1.077–5.170; *p* = 0.032). This study demonstrates that the evaluation of the functional status of CD8 + T cells in combination with the analysis of immunosuppressive elements could provide clinically relevant information in different breast cancer subtypes.

**Supplementary Information:**

The online version contains supplementary material available at 10.1007/s00262-024-03630-8.

## Introduction

Invasive breast carcinoma is the most commonly diagnosed malignancy in women worldwide with 2.26 million new cases in 2020 [1]. Despite advancements in diagnosis and treatment, breast cancer remains a leading cause of female cancer death. In routine practice, breast carcinoma is classified according to the status of four prognostic and predictive biomarkers, namely estrogen receptor (ER), progesterone receptor (PR), proliferation marker Ki67 and human epidermal growth factor receptor 2 (HER2/Neu) into luminal A, luminal B, HER2 positive (HER2 +) and triple negative (TNBC) [2].

Tumor-infiltrating lymphocytes (TILs) are gaining attraction as an emerging biomarker. In the literature, high CD8 + cytotoxic TILs are mainly related to improved prognosis and better treatment response to chemotherapy and immunotherapy [3–5]. The magnitude of TILs varies along breast cancer subtypes, with TNBC and HER2 + exhibiting a higher CD8 + T cell infiltration than luminal type cases explaining, at least partly, the improved response of TNBC to immunotherapy [6]. However, there are a few studies showing an inverse association between CD8 + cell count and prognosis or no statistical association between CD8 + T cell density and prognosis in breast cancer patients [7–9]. In addition, assessment of T cell count is not enough, and it may then be necessary to provide functional insight of CD8 + T cells in the tumor microenvironment (TME). Along this line, we demonstrated that increased infiltration by T cells in a subset of patients with pancreatic cancer adenocarcinoma does not necessary mean that this population is functional [10]. Thus, to obtain clinically relevant data, it is important to study the distinct functional T cell states.

Proliferating CD8 + T cells represent a state of activated T cells during the effector phase of the immune response that potentiates their effector function [11]. Little is known about the profile and the role of proliferating CD8 + T cells in TME. Studies in human tumors have demonstrated that TILs exhibit exhaustion features [12]. T cell exhaustion is a basket term representing an overall T cell hyporesponsive state observed in chronic infections and cancer [13]. Exhausted T cells are classified into two subsets: progenitor and terminally differentiated exhausted T cells [13]. Progenitor exhausted T cells have proliferating, memory, and cell stemness properties, while terminally differentiated exhausted loose these properties [13]. T cell exhaustion is linked with the expression of the transcription factor T cell factor 1 (TCF1, also known as *TCF7* by the gene encoded) [14]. TCF1 is expressed by progenitor T cells, while its expression is decreased in terminally exhausted T cells [13, 15]. TCF1 was initially examined in chronic viral diseases, demonstrating that TCF1 expression by CD8 + T cells is associated with improved viral disease control [14]. In cancer, available data in melanoma patients support that increased frequency of progenitor exhausted CD8 + TCF1 + T cells predicts response to immune checkpoint blockade [16, 17]. The expression of TCF1 by CD8 + TILs and its clinical impact in breast cancer patients remains unclear.

Besides, the interaction of T lymphocytes with other components of breast cancer microenvironment requires further delineation. In this sense, tumor-associated macrophages (TAMs) are important players in breast cancer microenvironment [18]. TAMs share an alternatively activated (also known as M2) pro-tumorigenic phenotype expressing the hemoglobin/haptoglobin receptor CD163 [19]. Increased CD163 + infiltration is associated in most studies with dismal prognosis in breast cancer patients [20, 21], although there is no consistency on the prognostic impact of CD163 + density among different breast cancer subtypes [22]. Interestingly, it was recently demonstrated that high CD8 + cytotoxic T cell density is associated with high CD163 + TAMs in invasive breast cancer patients suggesting an immune-intrinsic negative feedback mechanism [23].

TILs’ density is associated with programmed death 1 (PD-1) / programmed death ligand 1 (PD-L1) expression [24], further supporting that TILs’ assessment could guide treatment decision. Accordingly, PD-L1 expression is elevated in TNBC patients relative to other breast subtypes [25]. However, the landscape for PD-L1-based immunotherapy in breast cancer is still complex and not widely widespread. So far, anti-PD-L1 immunotherapy gained both Food and Drug Administration (FDA) and European Medicines Agency (EMA) approval only as neoadjuvant and maintenance adjuvant setting in high risk early TNBC as well as front line treatment in association with chemotherapy in patients with metastatic TNBC [26].

This current study aims to investigate the status and prognostic impact of distinct T cell states focusing on proliferating CD8 + T cells including TCF1-expressing tumor-infiltrating CD8 + cells in association with CD163 + and CD163 + PD-L1 + macrophages as well as PD-L1 status in a well-characterized cohort of treatment naïve breast cancer patients encompassing different breast cancer subtypes [27–29].

## Material and methods

### Study population

Breast tissue microarrays (TMAs) were generated from formalin-fixed paraffin-embedded (FFPE) tissues of a well-characterized cohort of previously described treatment-naïve breast cancer patients [27–29]. All patients were diagnosed at Seoul National University Hospital. Selected clinicopathological parameters with established prognostic significance (e.g., molecular subtype, tumor grade, stage, and presence/absence of lymphovascular invasion or lymph node metastasis) were retrieved from the patients’ electronic medical records and were additionally recorded. Patients who received neoadjuvant therapy were excluded from the study. A total of 791 patients encompassing 284 patients with luminal type A, 28 patients with luminal type B, 46 HER2 + patients and 433 TNBC patients with available tissue material from the primary tumor were included in the study. TMAs contained tissue from tumor center. TMAs were provided by the Seoul National University College of Medicine / Seoul National University Hospital and approved by the Institutional Review Board (IRB) (No. H-1512–076-728). This study was further approved by the Bioethics Committee of the Medical School of National and Kapodistrian University of Athens (Νο. 553/24.09.2021 & 152/27.06.2019), in accordance with the Declaration of Helsinki and local laws and regulations.

## Immunohistochemistry (IHC)

Double IHC was performed on FFPE TMAs. The following antibodies were used: anti-CD8 (108 M, Cell Marque, 1:60); anti-Ki67 (clone 8D5, Cell Signaling 1:150); anti-CD163 (NCL-CD163, Novocastra-Leica, 1:100); anti-PD-L1 (clone SP263, Roche); and anti-TCF1 (clone C63D9, Cell Signaling,1:50). The following double immunostainings were performed: CD8-Ki67, CD163-PD-L1, and CD8-TCF1. TMAs sections were counterstained with Mayer’s hematoxylin. To exclude background staining in double immunostainings, we omitted sequentially the primary antibody (Supplementary Fig. 1a–c). Human tonsil served as positive control for CD8, Ki67, TCF1, PD-L1, and human placenta for CD163 (Supplementary Fig. 1d). For CD8, TCF1, and CD163, we assessed the number of positive ( +) stromal cells per high power field (HPF, magnification 400x). We also assessed separately the percentage of TCF1 (%) of cancer cells. For the evaluation of PD-L1, we employed the Combined Positive Score (CPS) which is the number of PD-L1 + cancer cells, lymphocytes and macrophages divided by the total number of viable cancer cells, multiplied by 100 [30]. We further assessed separately the number of PD-L1 + stromal cells per HPF. For CD8-Ki67, CD163-PD-L1, and CD8-TCF1 assessment, we examined the number of CD8 + Ki67 + , CD163 + PD-L1 +, and CD8 + TCF1 + stromal cells per HPF.

## Bioinformatic analysis

An open-source web-based bioinformatic tool called the Kaplan–Meier plotter (http://kmplot.com/analysis/index.php?p=background) was employed, that includes gene chip and RNA-seq data by GEO, EGA, and TCGA, in order to assess the prognostic impact [31].

## Statistical analysis

Nonparametric methods were employed for all statistical analyses. In addition, all statistical tests were conducted to the significance level of alpha ≤ 0.05.

For every biomarker, we used the nonparametric Kruskal–Wallis test when we compared three or more groups (i.e., the luminal type A, luminal type B, HER2 + and TNBC subtypes). Upon significance of the Kruskal–Wallis’s test, post hoc pairwise comparisons of the 4 breast subtypes were performed using a Dunn test and the resulting p values were Holm-adjusted to account for multiple testing. When we compared two groups (i.e., stage I/II vs stage III/IV cancers), the Mann–Whitney’s U test was performed instead. The biomarker values were assessed graphically using boxplots displaying both the medians and the ranks of the single subtypes. Further, Spearman’s *ρ* correlations between the single biomarker types were calculated and displayed in a correlation matrix together with their significances. The p values were obtained based on a Spearman test and were Holm-adjusted for multiple testing.

To evaluate the impact of each biomarker expression on the disease-free survival (DFS), Kaplan–Meier (KM) curves in association with univariate and multivariate Cox regression analyses were performed. DFS was defined as the time of diagnosis until the date of documented disease relapse. Cutoff values were first determined with the *cutp()* function of the *survMisc* package (Supplementary Table 1) and then used to split the expression data of each biomarker in two groups (high vs low expression). The same cutoff values were used for all analyses. In the KM analysis, the survival curves of these groups were compared using the log-rank test. In addition to the whole cohort, survival analysis (both with KM curves and univariate/multivariate Cox regression) was separately conducted for the luminal type A and TNBC patients.

All analyses were performed using the statistical software R Version 4.2.3 (R Foundation for statistical computing Vienna, Austria).

## Results

### CD8 + T cell states with unique profile among breast cancer subtypes

**Fig. 1:**
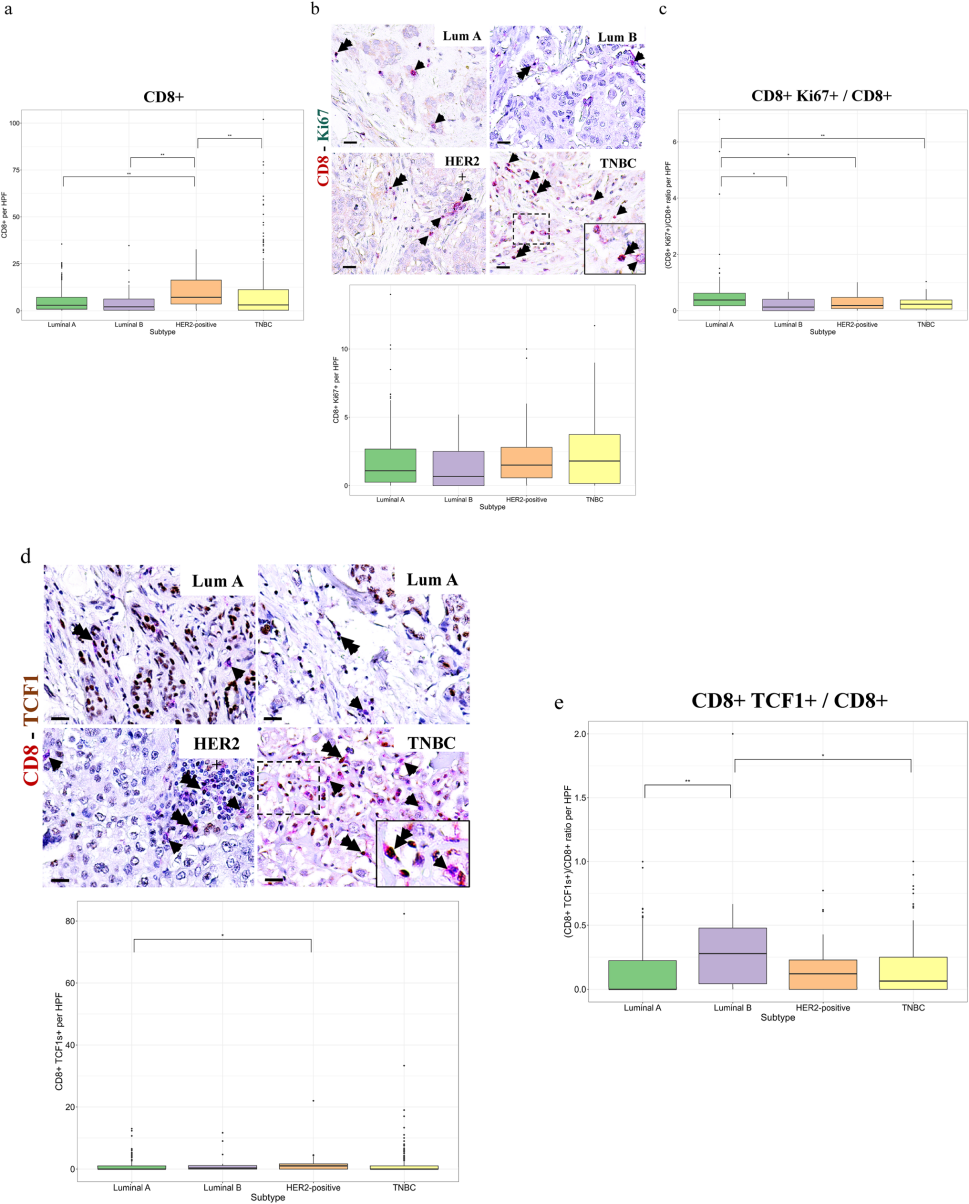
Assessment of proliferating CD8 + /TCF1 + T cells between breast cancer subtypes. **a** CD8 + T cell density was significantly increased in HER2 + and TNBC compared to luminal A and B tumors. **b** Profile of proliferating CD8 + T lymphocytes. Representative micrographs showing CD8 (red) and Ki67 (green) immunostaining along with corresponding quantification. Arrowheads indicate CD8 + T cells, and double arrowheads indicate CD8 + /Ki67 + T cells (scale bar: 50 μm). Insert refers to dashed area. **c** Ratio of proliferating CD8 + Ki67 + T cells. CD8 + Ki67 + / CD8 + were significantly decreased in TNBC and HER2 + compared to luminal A tumors. **d** Profile of CD8 + TCF1 + T lymphocytes. Representative micrographs showing TCF1 (brown) and CD8 (red) immunopositivity along with corresponding quantification. Arrowheads indicate CD8 + cells, and double arrowheads indicate CD8 + /TCF1 + T cells (scale bar: 50 μm). Insert refers to dashed area. **e** CD8 + TCF1 + / CD8 + status

CD8 + T cell density was significantly increased in HER2 + and to a lesser extent in TNBC breast cancer patients versus luminal A and B patients (Fig. 1a). We next asked for the state of CD8 + T lymphocytes in TME. Further characterization of CD8 + T lymphocytes co-expressing the proliferating marker Ki67 did not reveal significant differences between the different subtypes (Fig. 1b). However, assessment of CD8 + Ki67 + / CD8 + ratio revealed a significant decrease in the fraction of proliferating CD8 + T cells in TNBC and HER2 + compared to luminal type A tumors (Fig. 1c). Further evaluation of TCF1 expression by CD8 + TILs could not reveal significant differences between luminal A and TNBC tumors that comes in line with the status of CD8 + Ki67 + T cells (Fig. 1d); conversely infiltration by CD8 + TCF1 + cells was significantly elevated in HER2 + patients compared to luminal A tumors (Fig. 1d). Assessment of the ratio of CD8 + TCF1 + / CD8 + did not reveal significant differences between luminal A and TNBC tumors, while it was decreased in TNBC versus luminal B tumors (Fig. 1e). Interestingly, there was a significant increased presence of TCF1 + stromal cells, in both HER2 + and TNBC relative with luminal A tumors (Supplementary Fig. 2a). These data are presented cumulatively in Supplementary Table 2. To further characterize the cells expressing TCF1 + in the stroma, co-staining of CD4 + T cells along with TCF1 revealed that apart from CD8 + , CD4 + lymphocytes also frequently co-express TCF1 (Supplementary Fig. 2b). Stratification according to Tumor Node Metastasis (TNM) stage revealed that only for Stages I and II there were significant differences in tumor-infiltrating CD8 + , CD8 + TCF1 + , TCF1 + cells along with CD8 + Ki67 / CD8 + and CD8 + TCF1 + / CD8 + ratios among breast cancer subtypes (Supplementary Tables 3–5). As the luminal A and TNBC group of patients harbored the most cases, further analysis of the aforementioned biomarker’s expression between Stages I/II and III/IV did not reveal significant differences (Supplementary Tables 6, 7).

Since TCF1 is also involved in breast cancer biology [32], we assessed the status of TCF1 in cancer cells. The percentage of TCF1 + expressing cancer  cells [TCF1(%)] was significantly reduced in the TNBC versus luminal type A cases (Supplementary Fig. 2c). Interestingly, in the whole cohort, TCF1 (%) of cancer cells was significantly increased in stages I and II versus III and IV (Supplementary Table 5).

## Increased CD163 + , PD-L1 and CD163 + PD-L1 + cells in TNBC

**Fig. 2:**
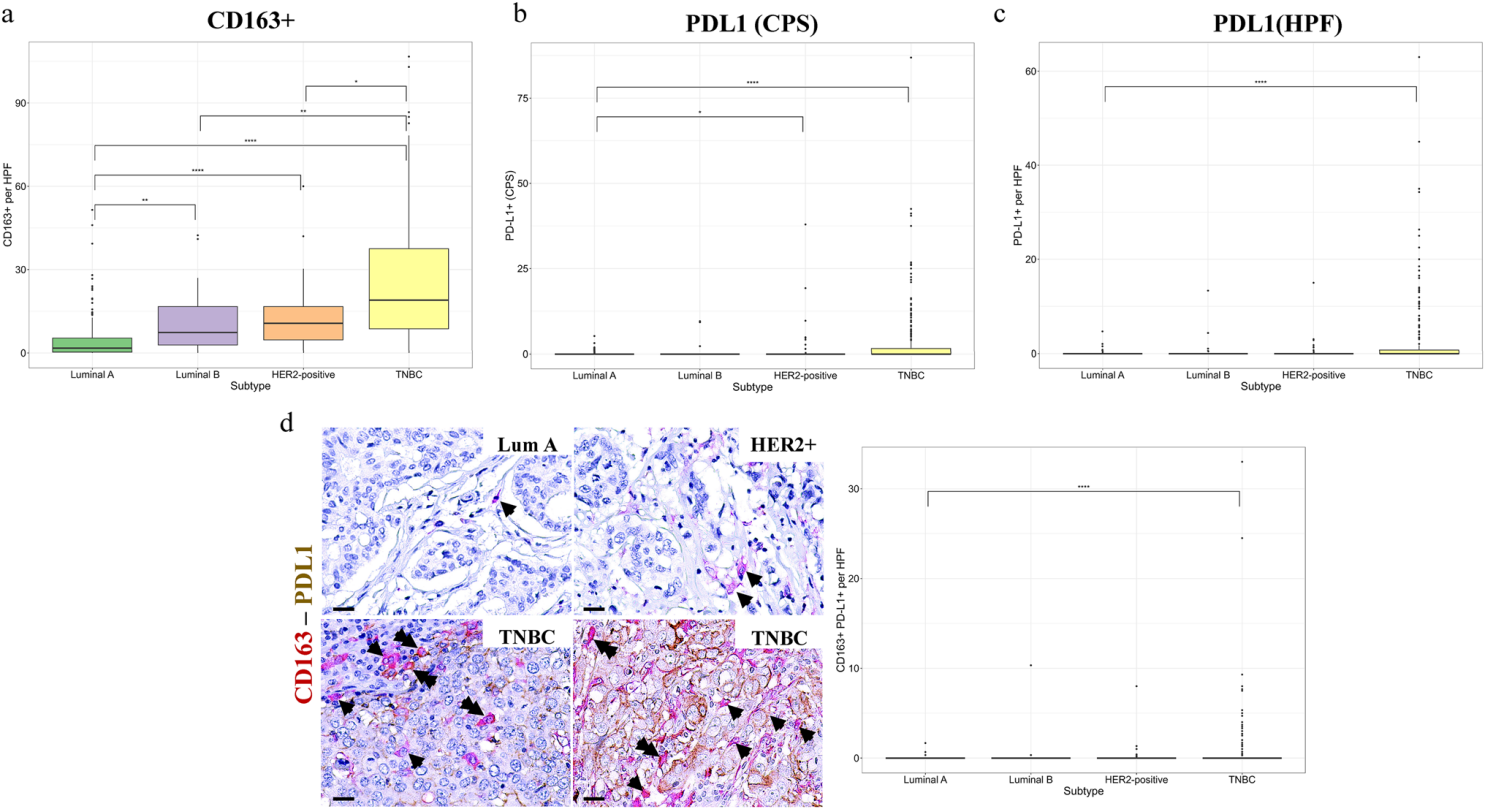
Assessment of infiltrating CD163 + /PD-L1 + macrophages along with PD-L1 status between breast cancer subtypes. **a** Quantification of CD163 + cells. CD163 + cells were significantly increased in TNBC and HER2 + compared to luminal tumors. **b** Quantification of PD-L1 CPS score. **c** Assessment of PD-L1 expression by stromal cells per high power field (HPF, magnification 400x). **d** Representative micrographs showing PD-L1 (brown) and CD163 (red) immunopositivity along with the corresponding quantification. Arrowheads indicate CD163 + macrophages, and double arrowheads indicate CD163 + cells co-expressing PD-L1 (scale bar: 20 μm). CD163 + PD-L1 + cells are significantly increased in TNBC compared to luminal tumors

The assessment of CD163 + TAMs revealed a significant higher infiltration by CD163 + macrophages of TNBC and HER2 + in comparison with luminal A and B tumors (Fig. 2a). The assessment of PD-L1 expression revealed: a) In most cases, there was no PD-L1 immunostaining in cancer and stromal cells, and b) among breast cancer subtypes, PD-L1 was mainly expressed by cancer and stromal cells in TNBC (Fig. 2b, c). Interestingly, 82 and 27 TNBC cases exhibited PD-L1 CPS score > 1 and > 10, respectively (Fig. 2b). Further evaluation of PD-L1 expression by CD163 + macrophages demonstrated an increased density of CD163 + PD-L1 + cells mainly in TNBC patients (Fig. 2d). These data are presented cumulatively in Supplementary Table 2. After stratification of PD-L1 status and CD163 + PD-L1 + macrophages by TNM stage, significant differences among breast cancer subtypes were identified only in stage I and II breast cancer patients; interestingly, infiltration of CD163 + macrophages exhibited significant differences irrespective of staging among cancer subtypes (Supplementary Tables 3–5). Besides, comparison of the biomarkers’ status between stages found increased PD-L1 (CPS) and CD163 + PD-L1 + macrophages in stages I/II versus stages III/IV in TNBC (Supplementary Tables 5–7).

## Distinct impact of intra-tumoral CD8 + T cell states on DFS among luminal type A and TNBC subtypes

**Fig. 3:**
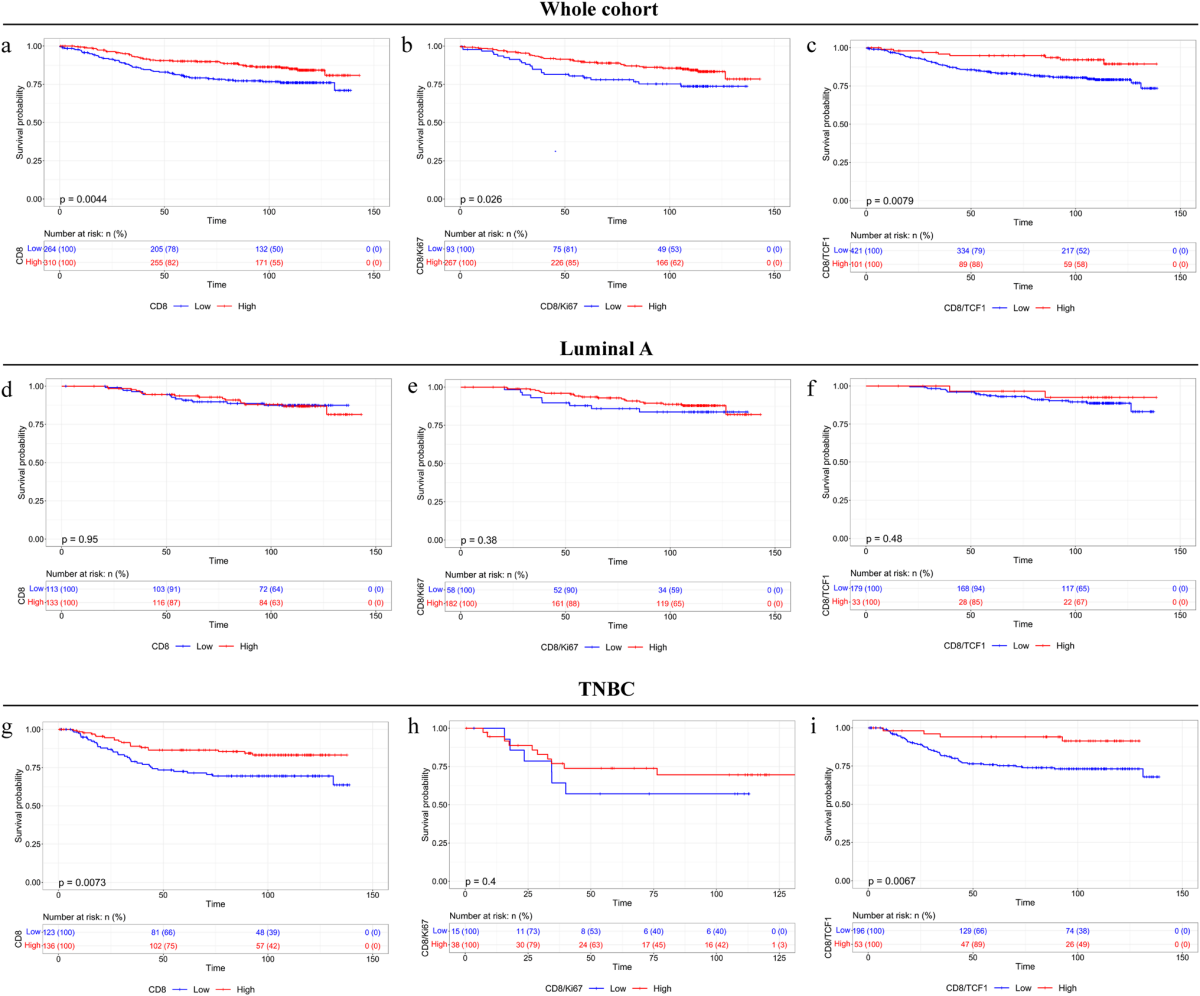
Kaplan–Meier survival analysis of CD8 + , CD8 + Ki67 + and CD8 + TCF1 + T cells in the whole population **a-c**, luminal A tumors **d-f** and TNBC **g-i**

Kaplan–Meier and univariate Cox regression survival analysis showed that for the whole group of patients there was a significant association between the increased CD8 + , CD8 + Ki67+ and CD8 + TCF1 + infiltration and the increased CD8 + Ki67 + / CD8 + ratio with longer DFS (Fig. 3a–c, Table 1). This was further verified employing bioinformatics analysis [31], integrating RNA-seq data from TCGA repository showing improved DFS in cases with increased CD8 + (*p* < 0.001) and TCF1/TCF7 + (*p* < 0.001) status (Supplementary Fig. 3a, b). No significant differences were observed with stromal TCF1 + and expression of TCF1 by cancer cells (Supplementary Fig. 3c, d). Notably, increased CD8 + Ki67 + / CD8 + was associated with improved DFS, while increased CD8 + TCF1 + / CD8 + was associated with dismal DFS (Table 1 and Supplementary Fig. 4a, b). Further Kaplan–Meier and univariate Cox regression survival analysis according to CD8 + T cell status were focused on luminal A and TNBC group of patients. Although the prognostic role of these biomarkers was not significant in luminal A tumors apart from CD8 + Ki67 / CD8 + ratio (Fig. 3d–f, Supplementary Fig. 4c-f and Supplementary Table 8), increased CD8 + and CD8 + TCF1 + T cell infiltration was associated with improved DFS in TNBC patients (Fig. 3g–i, Supplementary Fig. 4 g–j, Supplementary Table 10). Accordingly, increased TCF1 in the stroma is a favorable prognostic factor for DFS only in TNBC but not in luminal A tumors (Supplementary Fig. 4c, g). Assessment of TCF1 expression by cancer cells did not reveal any significant prognostic impact irrespective of breast cancer subtype (Supplementary Fig. 4d, h).

**Table 1:** Univariate Cox regression analysis for DFS in the whole cohort of breast cancer patients, evaluating the prognostic impact of the biomarkers tested

Biomarker	HR	95% CI	*P* value
CD8 + per HPF	0.567	0.382–0.842	**0.0049**
CD8 + Ki67 + per HPF	0.561	0.335–0.940	**0.028**
CD163 + per HPF	2.357	1.380–4.025	**0.0017**
PD-L1 + (CPS)	0.497	0.251–0.986	**0.046**
PD-L1 + per HPF	0.500	0.242–1.029	0.06
CD163 + PD-L1 + per HPF	0.401	0.163–0.986	**0.046**
TCF1s + per HPF	0.727	0.470–1.125	0.153
CD8 + TCF1s + per HPF	0.387	0.187–0.801	**0.01**
TCF1 (%) of cancer cells	0.564	0.292–1.089	0.088
(CD8 + Ki67 +)/CD8 + ratio per HPF	0.428	0.225–0.812	**0.0095**
(CD8 + TCF1s +)/CD8 + ratio per HPF	1.885	1.140–3.118	**0.014**

Significant *P* values (< 0.05) linked with the biomarkers’ expression are highlighted in bold

HR, hazard ratio; DFS, disease-free survival; CPS, combined positive score; and HPF, high power field

Multivariate Cox regression analysis adjusted for tumor stage [33], histological grade [34], and lymphovascular invasion [35], which are established prognostic markers in invasive breast cancer, revealed that CD8 + [hazard ratio (HR) = 0.607; 95% confidence interval (CI) 0.404–0.911; *p* = 0.016] and CD8 + TCF1 + (HR = 0.401; 95% CI 0.193–0.833; *p* = 0.014) tumor-infiltrating cells per HPF as well as CD8 + Ki67 + / CD8 + (HR = 0.429; 95% CI 0.219–0.838; *p* = 0.013) and CD8 + TCF1 + / CD8 + (HR = 1.805; 95% CI 1.084–3.006; *p* = 0.023) are independent prognostic biomarkers linked with DFS (Table 2). Multivariate analysis in TNBC cases further demonstrated that increased CD8 + (HR = 0.542; 95% CI 0.309–0.950; *p* = 0.032) and CD8 + TCF1 + (HR = 0.280; 95% CI 0.101–0.779; *p* = 0.015) cells per HPF are independently associated with a reduced patient relapse rate (Table 3). In addition, multivariate analysis in the luminal type A patient cohort revealed that increased CD8 + Ki67 + / CD8 + was linked with a lower relapse rate (HR = 0.405; 95% CI 0.169–0.974; *p* = 0.043) (Supplementary Table 9).

**Table 2:** Multivariate Cox regression analysis for DFS in the whole cohort of breast cancer patients, evaluating the prognostic impact of each biomarker (separate model for each biomarker, adjusting each time for following covariates: tumor grade, stage, and presence of lymphovascular invasion)

Biomarker	Variable	HR	95% CI	*P* value
CD8 + per HPF	CD8 + per HPF	0.607	0.404–0.911	**0.016**
	Grade (III vs. I/II)	2.231	1.352–3.683	0.0017
	Stage (III/IV vs. I/II)	1.605	0.990–2.603	0.055
	Lymphovascular invasion	1.656	1.088–2.522	0.019
CD8 + Ki67 + per HPF	CD8 + Ki67 + per HPF	0.632	0.370–1.080	0.093
	Grade (III vs. I/II)	3.074	1.614–5.852	< 0.001
	Stage (III/IV vs. I/II)	1.599	0.843–3.033	0.151
	Lymphovascular invasion	1.308	0.763–2.242	0.329
CD163 + per HPF	CD163 + per HPF	1.767	1.014–3.077	**0.044**
	Grade (III vs. I/II)	1.831	1.082–3.097	0.024
	Stage (III/IV vs. I/II)	1.941	1.210–3.114	0.0059
	Lymphovascular invasion	1.644	1.076–2.512	0.021
PD-L1 + (CPS)	PD-L1 + (CPS)	0.431	0.215–0.866	**0.018**
	Grade (III vs. I/II)	2.433	1.448–4.089	< 0.001
	Stage (III/IV vs. I/II)	1.846	1.149–2.967	0.011
	Lymphovascular invasion	1.512	0.988–2.313	0.057
PD-L1 + per HPF	PD-L1 + per HPF	0.422	0.202–0.879	**0.021**
	Grade (III vs. I/II)	2.434	1.448–4.090	< 0.001
	Stage (III/IV vs. I/II)	1.861	1.158–2.991	0.01
	Lymphovascular invasion	1.516	0.991–2.320	0.055
CD163 + PD-L1 + per HPF	CD163 + PD-L1 + per HPF	0.357	0.144–0.886	**0.026**
	Grade (III vs. I/II)	2.356	1.406–3.949	0.0011
	Stage (III/IV vs. I/II)	1.859	1.158–2.984	0.01
	Lymphovascular invasion	1.540	1.009–2.352	0.045
TCF1s + per HPF	TCF1s + per HPF	0.652	0.414–1.027	0.065
	Grade (III vs. I/II)	2.524	1.439–4.426	0.0012
	Stage (III/IV vs. I/II)	1.704	1.024–2.836	0.04
	Lymphovascular invasion	1.600	1.012–2.531	0.044
CD8 + TCF1s + per HPF	CD8 + TCF1s + per HPF	0.401	0.193–0.833	**0.014**
	Grade (III vs. I/II)	2.366	1.362–4.111	0.0022
	Stage (III/IV vs. I/II)	1.648	0.994–2.732	0.053
	Lymphovascular invasion	1.628	1.041–2.547	0.033
TCF1 (%) of cancer cells	TCF1 (%) per HPF	0.719	0.370–1.397	0.33
	Grade (III vs. I/II)	2.236	1.285–3.893	0.0044
	Stage (III/IV vs. I/II)	1.643	0.987–2.734	0.056
	Lymphovascular invasion	1.710	1.094–2.671	0.018
(CD8 + Ki67 +)/CD8 + ratio per HPF	(CD8 + Ki67 +)/CD8 + ratio per HPF	0.429	0.219–0.838	**0.013**
	Grade (III vs. I/II)	2.405	1.242–4.656	0.0093
	Stage (III/IV vs. I/II)	1.967	1.015–3.813	0.045
	Lymphovascular invasion	1.324	0.750–2.336	0.333
(CD8 + TCF1s +)/CD8 + ratio per HPF	(CD8 + TCF1s +)/CD8 + ratio per HPF	1.805	1.084–3.006	**0.023**
	Grade (III vs. I/II)	2.683	1.394–5.165	0.0031
	Stage (III/IV vs. I/II)	1.460	0.820–2.599	0.198
	Lymphovascular invasion	1.880	1.142–3.093	0.013

Significant *P* values (< 0.05) linked with the biomarkers’ expression are highlighted in bold

HR, hazard ratio; DFS, disease-free survival; CPS, combined positive score; and HPF, high power field

**Table 3:** Multivariate Cox regression analysis for DFS in the TNBC patients, evaluating the prognostic impact of each biomarker (separate model for each biomarker, adjusting each time for the following covariates: tumor grade, stage, and presence of lymphovascular invasion)

Biomarker	Variable	HR	95% CI	*P* value
CD8 + per HPF	CD8 + per HPF	0.542	0.309–0.950	**0.032**
	Grade (III vs. I/II)	1.332	0.598–2.965	0.482
	Stage (III/IV vs. I/II)	1.729	0.948–3.153	0.074
	Lymphovascular invasion	1.714	0.956–3.075	0.071
CD163 + per HPF	CD163 + per HPF	1.291	0.398–4.195	0.67
	Grade (III vs. I/II)	1.342	0.602–2.996	0.472
	Stage (III/IV vs. I/II)	2.130	1.179–3.849	0.012
	Lymphovascular invasion	1.691	0.935–3.057	0.082
PD-L1 + (CPS)	PD-L1 + (CPS)	0.362	0.162–0.812	**0.014**
	Grade (III vs. I/II)	1.570	0.703–3.505	0.271
	Stage (III/IV vs. I/II)	1.965	1.088–3.547	0.025
	Lymphovascular invasion	1.539	0.856–2.767	0.15
PD-L1 + per HPF	PD-L1 + per HPF	0.395	0.176–0.884	**0.024**
	Grade (III vs. I/II)	1.536	0.688–3.428	0.295
	Stage (III/IV vs. I/II)	2.005	1.111–3.619	0.021
	Lymphovascular invasion	1.549	0.861–2.786	0.144
CD163 + PD-L1 + per HPF	CD163 + PD-L1 + per HPF	0.312	0.112–0.870	**0.026**
	Grade (III vs. I/II)	1.485	0.666–3.311	0.334
	Stage (III/IV vs. I/II)	1.943	1.077–3.505	0.027
	Lymphovascular invasion	1.640	0.914–2.940	0.097
TCF1s + per HPF	TCF1s + per HPF	0.608	0.340–1.087	0.093
	Grade (III vs. I/II)	1.384	0.617–3.103	0.43
	Stage (III/IV vs. I/II)	1.813	0.971–3.386	0.062
	Lymphovascular invasion	1.631	0.876–3.037	0.123
CD8 + TCF1s + per HPF	CD8 + TCF1s + per HPF	0.280	0.101–0.779	**0.015**
	Grade (III vs. I/II)	1.330	0.597–2.963	0.485
	Stage (III/IV vs. I/II)	1.744	0.947–3.212	0.074
	Lymphovascular invasion	1.751	0.968–3.168	0.064
TCF1 (%) of cancer cells	TCF1 (%) per HPF	1.137	0.451–2.869	0.785
	Grade (III vs. I/II)	1.299	0.582–2.902	0.523
	Stage (III/IV vs. I/II)	1.696	0.913–3.148	0.094
	Lymphovascular invasion	1.824	1.004–3.315	0.049
(CD8 + TCF1s +)/CD8 + ratio per HPF	(CD8 + TCF1s +)/CD8 + ratio per HPF	1.499	0.765–2.937	0.238
	Grade (III vs. I/II)	2.702	0.647–11.285	0.173
	Stage (III/IV vs. I/II)	1.425	0.698–2.913	0.331
	Lymphovascular invasion	2.280	1.158–4.490	0.017

Significant *P* values (< 0.05) linked with the biomarkers’ expression are highlighted in bold

For the biomarkers “CD8 + Ki67 + per HPF” and “(CD8 + Ki67 +)/CD8 + ratio per HPF,” the number of events was small to run a multivariable Cox regression model

HR, hazard ratio; DFS, disease-free survival; CPS, combined positive score; and HPF, high power field

## Distinct impact of CD163 + , PD-L1 +, and CD163 + PD-L1 + on DFS among luminal type A and TNBC tumors

**Fig. 4:**
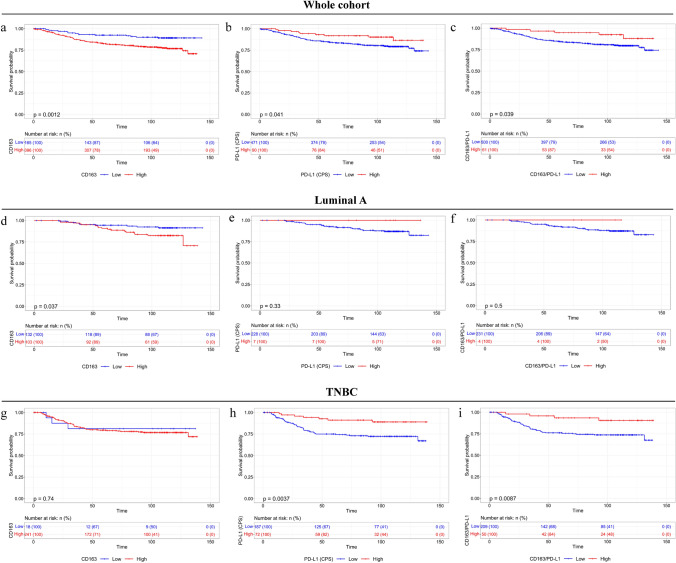
Kaplan–Meier survival analysis of CD163 + cells, PD-L1 (CPS), and CD163 + PD-L1 + cells in the whole population **a-c**, luminal A tumors **d-f,** and TNBC **g-i**

The impact of CD163 + , PD-L1 +, and CD163 + / PD-L1 + cells on DFS revealed that increased infiltration by CD163 + is a dismal prognostic factor in the whole population (Fig. 4a, Table 1). On the other hand, an increased PD-L1 expression and infiltration by CD163 + PD-L1 + cells were associated with favorable DFS (Fig. 4b, c, Supplementary Fig. 5a, Table 1). These findings were further verified employing KM plotter (Supplementary Fig. 5b). Increased CD163 expression was associated with dismal DFS only in patients with luminal type A but not in TNBC tumors (Fig. 4d, g, Table 1). Conversely, an increased PD-L1 score as well as increased infiltration by PD-L1 + and CD163 + PD-L1 + cells was associated with a significantly better DFS only in TNBC compared to luminal A tumors (Fig. 4e, f, h, i and Supplementary Fig. 6a, b, Supplementary Tables 8 and 10).

Multivariate Cox regression analysis demonstrated that high CD163 + (HR: 1.767; 95% CI 1.014–3.077; p = 0.044) is independent prognostic factor linked with a higher relapse rate, whereas increased CD163 + PD-L1 + (HR: 0.357; 95% CI 0.144–0.886; *p* = 0.026) macrophages as well as increased PD-L1 + (CPS) (HR: 0.431; 95% CI 0.215–0.866; *p* = 0.018) and PD-L1 + per HPF (HR: 0.422; 95% CI 0.202–0.879; *p* = 0.021) are independent prognostic factors linked with a lower relapse rate (Table 2). Further, multivariate analysis in TNBC patients depicted that increased CD163 + PD-L1 + (HR: 0.312; 95% CI 0.112–0.870; *p* = 0.026) macrophages, PD-L1 + (CPS) (HR: 0.362; 95% CI 0.162–0.812; *p* = 0.014), and PD-L1 + per HPF (HR: 0.395; 95% CI 0.176–0.884; *p* = 0.024) are independently linked with a lower relapse rate (Table 3). Furthermore, multivariate analysis in the Luminal type A patient cohort revealed that increased CD163 + was associated with a higher relapse rate (HR = 2.360; 95% CI 1.077–5.170; *p* = 0.032) (Supplementary Table 9).

**Fig. 5:**
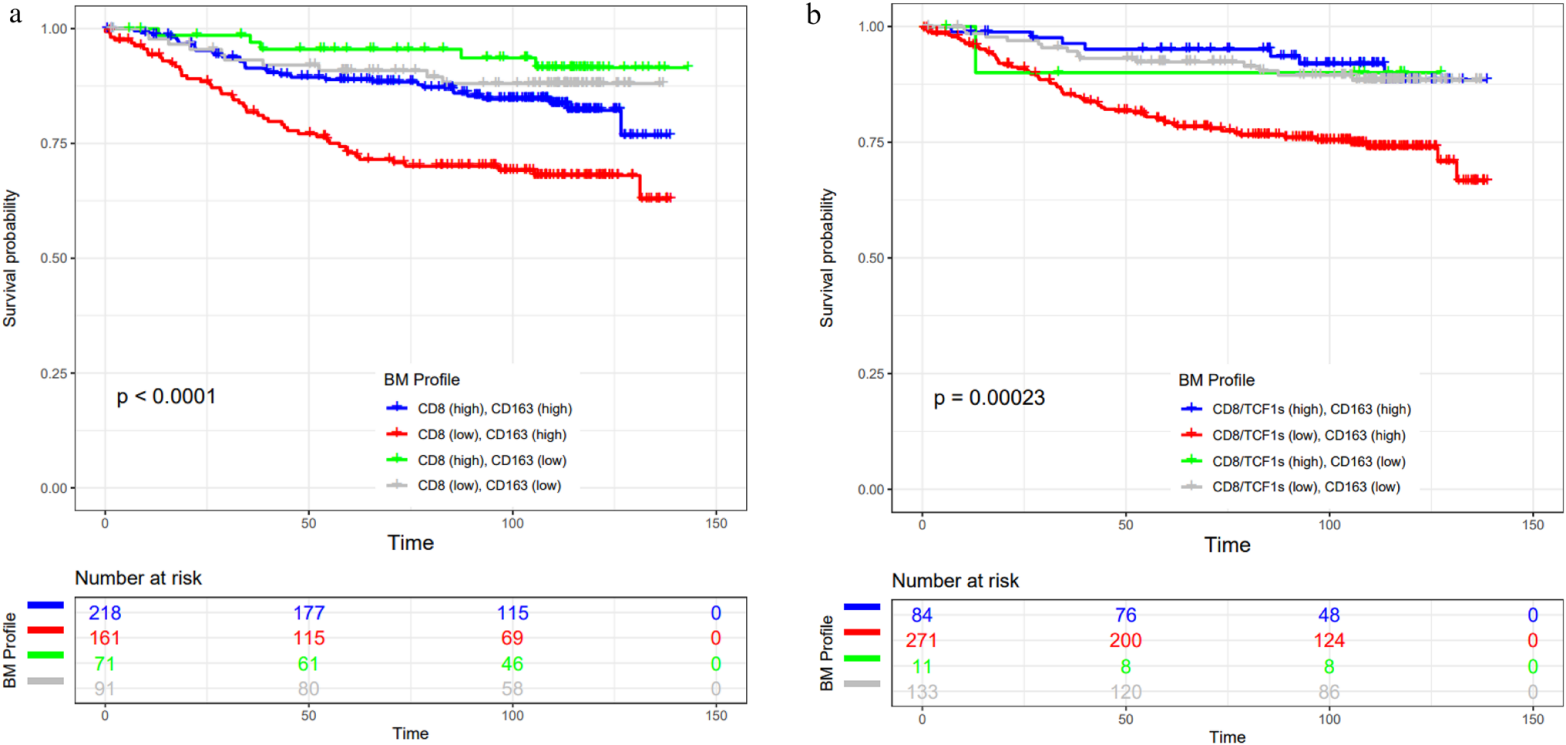
Combined analysis in the whole population associating CD8 + and CD163 + cells **a** as well as CD8 + TCF1 + and CD163 + cells **b** with DFS

Combined analysis demonstrated that breast cancer patients with CD8 (low) and CD163 (high) as well as CD8 + TCF1 + (low) and CD163 (high) levels were associated with worse DFS (*p* < 0.0001 and *p* = 0.00023, respectively; Fig. 5a, b). Further combined assessment of these parameters retained a persistent prognostic value among luminal A and TNBC patients. Indeed, although no significant associations were found in luminal A patients (Supplementary Fig. 7a), in TNBC patients CD8 (low) and CD163 (high) were associated with worse DFS (p = 0.028) (Supplementary Fig. 7b).

## Association between CD8 + T cell states with CD163 and PD-L1

**Fig. 6:**
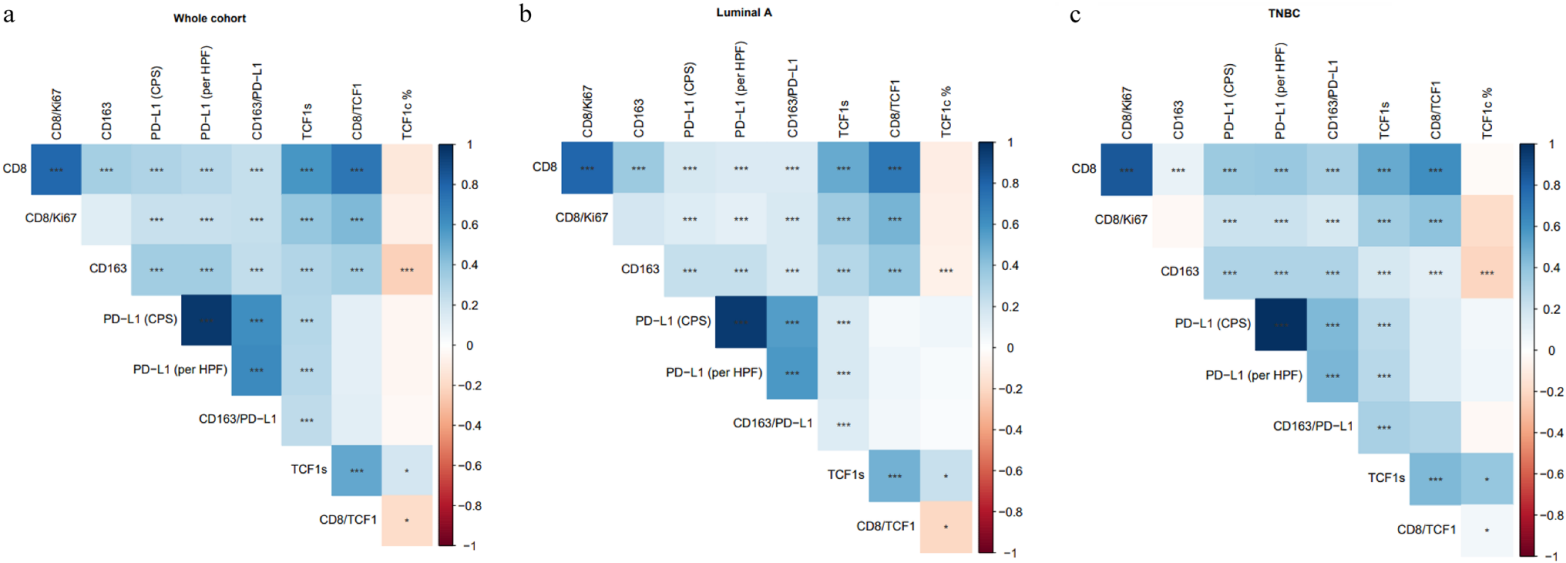
Association between distinct CD8 + T cell states with CD163 + cells and PD-L1 status as well as with TCF1 expression by cancer cells in the whole population **a**, luminal A tumors **b**, and TNBC **c**

Prompted by a recent study showing a direct correlation between CD8 + and CD163 + in breast cancer patients [23], we observed that increased CD8 + , CD8 + Ki67 +, and CD8 + TCF1 + density was associated with high PD-L1 score, CD163 +, and infiltration by CD163 + PD-L1 + cells, irrespective of the breast cancer subtype (Fig. 6a, b, c and Supplementary Fig. 8a, b). These findings show that high magnitude of TILs is related to increased density of cells with an immune suppressor phenotype. Interestingly, there was an inverse association between infiltration by CD163 + macrophages and TCF1 expression by cancer cells in luminal A, TNBC and HER2 + tumors (Fig. 6b, c and Supplementary Fig. 8b).

## Discussion

To improve breast cancer patient stratification and selection for immune therapy-based approaches, it is necessary to discover new biomarkers with prognostic and predictive value. Thus, there is an urgent need to understand the immune landscape in the breast cancer microenvironment. As CD8 + T cells and myeloid cells make a large part of inflammatory infiltrate in TME, in this study we assessed the state of CD8 + T cells along with CD163 + macrophages and PD-L1 status in a well-characterized cohort of 791 treatment-naïve breast cancer patients [27–29].

We confirmed the increased infiltration by CD8 + TIL in HER2 + and TNBC breast cancer compared to patients with luminal subtypes as previously reported [6] (Fig. 1). Indeed, previous studies have shown that tumors with high tumor mutational burden (TMB), a measure of the rate of non-synonymous mutations per DNA megabase, are associated with T cell rich TME [36]. Tumors with high TMB express more neoantigens and thus are more immunogenic, which in turn enhances T cell recruitment in the tumor bed [36]. In accordance, patients with HER2 + tumors and TNBC have the highest TMB, while luminal type A tumors have the lowest one [37] that comes in line with the high CD8 + T cell count in our TNBC and HER2 + cohorts.

Of note, our study demonstrated that the ratio of CD8 + Ki67 + / CD8 + cells in TNBC was significantly decreased versus luminal type cases (Fig. 1). Similarly, the ratio of proliferating CD8 + T cells was decreased in HER2 + compared to luminal breast cancer patients. In principle, proliferating TILs reflect an ongoing immune response of the host [38], and thus, the decreased infiltration by proliferating CD8 + T cells in TNBC patients strongly suggests that T cell response is compromised. This finding becomes even more interesting taking into consideration the increased T cell count in breast cancer patients harboring TNBC and HER2 + tumors. Hence, although there is marked T cell recruitment in TNBC and HER2 + TME, this is not accompanied by increased proliferative activity of infiltrating T cells.

The current study failed to demonstrate any significant difference in the status of CD8 + TCF1 + cells between luminal A tumors and TNBC (Fig. 1). A putative explanation of this observation could be attributed to the heterogeneity of T cells infiltrating TME [12]. Indeed, in the present study TCF1 expression by stromal cells increases in patients with TNBC compared to patients with luminal A tumors (Supplementary Fig. 2). Our observation that TCF1 is also expressed by CD4 + T cells clearly supports that further analysis is needed to better characterize the various states of TILs in breast cancer.

An interesting finding in this study was the decreased TCF1 expression by cancer cells in the TNBC and HER2 + tumor subtypes compared to luminal A tumors (Supplementary Fig. 2C). TCF1 is a downstream effector of Wingless/Integration 1 (Wnt)–β-catenin signaling pathway [14]. Activated Wnt signaling promotes breast carcinogenesis by inducing proliferation, invasion, and metastasis [39]. Notably, aberrant activation of Wnt pathway is associated with TNBC [39], which could explain at least in part our findings. As the expression of TCF1 by cancer cells is still poorly investigated, additional analysis is required. In addition, the observed inverse association between infiltrating CD163 + cells and TCF1 expression by cancer cells needs to be further investigated.

In the present study, we demonstrated a similar pattern of CD8 + and the suppressor CD163 + cells among the different breast cancer subtypes (Fig. 2). Notably, high CD8 + cell density in TNBC and HER2 + tumors are associated with increased count of CD163 + cells (Fig. 6). These findings come in line with a previous study showing a similar association between CD8 + and CD163 + cell densities in breast cancer patients, even though the limited number of TNBC patients precluded a definitive discrimination among the different breast cancer subtypes [23]. Similarly, PD-L1 status was significantly increased in TNBC and HER2 + versus luminal type A tumors (Fig. 2), in agreement with previous studies [25]. Accordingly, CD163 + TAMs co-expressed PD-L1 with a higher count in TNBC than luminal type A tumors (Fig. 2). A previous study also reported in a small number of TNBC patients (*n* = 20), an increased infiltration by CD163 + PD-L1 + cells [40]. Along this line, another  study demonstrated that PD-L1 expression by macrophages attenuates macrophage-mediated antitumor activity, while treatment with PD-L1 blocking antibodies reverses this phenotype triggering macrophage-mediated antitumor activity, stressing out the importance to study the immunophenotypic profile of TAMs [41]. Importantly, these findings suggest that immunologically “hot” tumors trigger endogenous immune suppressor pathways including increased recruitment of alternatively activated macrophages as well as elevated PD-L1 expression both by cancer and immune cells infiltrating TME. This may provide an explanation for the paradoxical relationship between increased PD-L1 score and increased CD163 + PD-L1 + status with improved DFS.

Our study has the following clinical implications: (i) Breast cancer treatment-naïve patients with high CD8 + , CD8 + Ki67 + , CD8 + TCF1 + , PD-L1 score are likely to experience improved DFS; in contrast, it is interesting to note despite the fact that high CD163 + cell infiltration per se was associated with decreased DFS, the patients whose tumors had a high CD163 + PD-L1 + cell infiltration presented with an improved DFS (Figs. 3, 4 and 5, Table 1). Our findings are in line with previous studies associating high CD8 + with low CD163 + count with favorable DFS in breast cancer patients [5, 42]; (ii) we demonstrated the differential impact of proliferating CD8 + /TCF1 + T cells as well as CD163 + infiltrating cells and PD-L1 status on DFS between luminal A and TNBC tumors. To the best of our knowledge, our study is the first one to be focused on the differential prognostic value of CD8 + Ki67 + , CD8 + TCF1 + , CD163 + PD-L1 + among luminal type A and TNBC tumors. Besides, this is the first study showing that proliferating CD8 + /TCF1 + T cells and CD163 + /PD-L1 + macrophages are independent prognostic markers for DFS in patients with breast carcinoma (Table 2, 3, Supplementary Table 9). A previous study examined the impact of proliferating CD8 + T cells in breast, colorectal, ovarian, gastric, pancreatic, and renal cell carcinomas and found that only colorectal cancer patients with elevated CD8 + /Ki67 + density are linked with favorable overall survival (OS), while increased proliferating CD8 + T cells is associated with poor prognosis in renal cell carcinomas [43]. No associations between CD8 + /Ki67 + cell density and OS in breast cancer patients were found, although the impact on DFS was not assessed [43]. Besides, this analysis did not evaluate the differential impact of proliferating CD8 + T cells between the different subtypes. To this end, as progenitor exhausted TCF1 + cells have stem cell-like properties and are likely responsible for the proliferative and functional immune responses that occur following immunotherapy [13], it is therefore not surprising that increased CD8 + TCF1 + is associated with improved DFS in breast cancer. Regarding CD163 status, our findings are in line with a previous study assessing TMAs from 144 patients, demonstrating that dense infiltration of CD163 + cells in the stroma is associated with dismal OS in luminal A but not TNBC patients [20]. Another study assessed the prognostic impact of CD163 in 398 breast cancer patients and came to a different conclusion, demonstrating that high CD163 + cell count is associated with improved OS in TNBC but not in luminal tumors [22]. This discrepancy could be attributed to technical issues including definitions, assessment protocol, and experimental procedure. In addition, similar conflicting results have been reported regarding the prognostic value of PD-L1 [30, 44]. In the current study, the assessment of PD-L1 was based on both the evaluation of CPS score and PD-L1 expression in stromal cells reaching to the same conclusions. Our data clearly demonstrated that elevated PD-L1 immunostaining is associated with improved DFS only in TNBC as previously has been reported [45]. Likewise, we recently demonstrated that high PD-L1 expression both in cancer cells and immune cells in TNBC is associated with a favorable prognosis supporting the significance of evaluating PD-L1 status in cancer cells and immune cells in this setting [46]. Notably, the combined assessment of CD8 + , CD8 + TCF1 + T cells along with CD163 + macrophages, and PD-L1 score may stratify luminal type A and TNBC patients in different risks for relapse groups.

A potential limitation of our study is that it is based on TMAs, which may be associated with over- or under-representation of the various immune cells’ markers due to tumor heterogeneity. However, the comparable results with the literature support the validity of our findings. Besides, in the literature analysis of TME is often based on TMA format, when a large number of samples are analyzed. Another limitation is that TCF1 expression was not examined simultaneously with one of the other markers expressed by exhausted T cells. Assessment of additional markers (like the inhibitory immune checkpoint receptor, PD-1) could improve our understanding of the distinct T cell exhaustion states. Notably, a recent study employing single-cell RNA sequencing analysis revealed that expression of PD-1 and CXCL13 on T cells and MHC-I (major histocompatibility complex I) on cancer cells could classify luminal breast cancer patients with respect to T cell exhaustion phenotype [47], denoting the complexity of studying T cell exhaustion in TME. An additional limitation is that CD8 is also expressed by other immune cell populations apart from T cell cytotoxic lymphocytes, including natural killer (NK)-like cells γδ T cells [48]. Furthermore, even though our cohort included treatment-naïve breast cancer patients that underwent surgical removal of their tumor, subsequent treatments likely affected patient prognosis. However, as information regarding the exact therapeutic strategy after surgery was largely not available, we could not assess whether the markers examined in this study have an impact on prognosis in association with different adjuvant therapy strategies, which is a limitation of the study. Besides, the cohort encompasses a large proportion of TNBC cases. To address this issue, we assessed separately luminal type A and TNBC patients.

Taken together, this study demonstrates a unique immunophenotypic profile of proliferating CD8 + including CD8 + TCF1 + T cells in combination with CD163 + infiltrating macrophages and PD-L1 status which could predict recurrence differently in luminal A and TNBC tumors. These findings highlight the importance of providing insight in the functional state of TILs rather than merely assessing their number. Besides, as TME is highly complex, to improve our understanding of immune landscape and to develop clinically relevant biomarkers, further studies combining TILs along with immune suppressive elements are necessary.

### Supplementary Information

Below is the link to the electronic supplementary material.

Supplementary file1: **Supplementary Figure 1**. Double immunohistochemistry in serial sections employing positive control (tonsil) for CD163+PD-L1+ and CD8+TCF1+. To exclude background staining in double immunostainings, we omitted sequentially the primary antibody (a, b) as well as both primary antibodies (c). Human placenta served as positive control for CD163 immunostaining (d). Scale bar: 100μm.**Supplementary Figure 2**. a Quantification of stromal TCF1+ cells among breast cancer subtypes. The density of stromal TCF1+ cells was significantly increased in HER2+ and TNBC compared to luminal tumors. b Representative micrographs showing TCF1 (brown) and CD4 (red) immunopositivity in luminal A tumors and TNBC. Arrowheads indicate TCF1+ cells and double arrowheads indicate TCF1+/CD4+ cells (scale bar: 50μm). c Quantification of the assessment of TCF1 (%) expression by cancer cells, showing significantly decreased expression in TNBC versus luminal A tumors.**Supplementary Figure 3**. a-b Kaplan–Meier survival curve employing KM plotter demonstrate that increased CD8A and TCF7 mRNA is associated with improved disease-free survival in breast cancer. c-d Kaplan–Meier survival analysis of TCF1s (stromal cells expressing TCF1) and TCF1c(%) (percentage of TCF1 expression by cancer cells) on the whole population. **Supplementary Figure 4**. Kaplan–Meier survival analysis of CD8+Ki67+ / CD8+ (a) and CD8+TCF1+ / CD8+ (b) on whole cohort. TCF1s (stromal cells expressing TCF1) and TCF1c(%) (percentage of TCF1 expression by cancer cells) on luminal A tumors (c, d) and TNBC (e, f). **Supplementary Figure 5**. a Kaplan–Meier survival analysis of PD-L1 status [assessment of PD-L1+ immune cells in the tumor microenvironment per high power field (HPF, magnification 400x)] in the whole population. b Kaplan–Meier survival curve employing KM plotter demonstrate that increased CD163 mRNA predicts poor disease-free survival in breast cancer. **Supplementary Figure 6**. Kaplan–Meier survival analysis of PD-L1 status [assessment of PD-L1+ immune cells in the tumor microenvironment per high power field (HPF, magnification 400x)] in luminal A tumors (a) and TNBC (b). **Supplementary Figure 7**. Combined analysis associating CD8+ and CD163+ cells as well as CD8+TCF1+ and CD163+ cells in luminal A (a) and TNBC (b) tumors. Supplementary Figure 8. Association between distinct CD8+ T cell states with CD163+ cells and PD-L1 status as well as with TCF1 expression by cancer cells in luminal B (a) and HER2+ (b) tumors. (PPTX 17043 kb)

Supplementary file2 (DOCX 48 kb)

## Data Availability

The data generated in this study are available within the article and its supplementary data file.

## Abbreviations

CI: Confidence interval
CPS: Combined positive score
DFS: Disease-free survival
ER: Estrogen receptor
HER2: Human epidermal growth factor receptor 2 (HER2/Neu)
HR: Hazard ratio
Lum: Luminal type
MHC-I: Major histocompatibility complex I
OS: Overall survival
PD-1: Programmed death 1
PD-L1: Programmed death ligand 1
PR: Progesterone receptor
TCF1: T cell factor 1
TILs: Tumor-infiltrating lymphocytes
TMAs: Tissue microarrays
TMB: Tumor mutational burden
TME: Tumor microenvironment
TNBC: Triple-negative breast cancer
TNM: Tumor Node Metastasis
