# Supplementary material for: Distinct profiles of proliferating CD8+/TCF1+ T cells and CD163+/PD-L1+ macrophages predict risk of relapse differently among treatment-naïve breast cancer subtypes
Source: Cancer Immunol Immunother. 2024 Feb 13;73(3):46. doi: 10.1007/s00262-024-03630-8 (PMC10864422; doi:10.1007/s00262-024-03630-8)
Supplement: Supplementary file 1 — Supplementary file1: Supplementary Figure 1. Double immunohistochemistry in serial sections employing positive control (tonsil) for CD163+PD-L1+ and CD8+TCF1+. To exclude background staining in double immunostainings, we omitted sequentially the primary antibody (a, b) as well as both primary antibodies (c). Human placenta served as positive control for CD163 immunostaining (d). Scale bar: 100μm.Supplementary Figure 2. a Quantification of stromal TCF1+ cells among breast cancer subtypes. The density of stromal TCF1+ cells was significantly increased in HER2+ and TNBC compared to luminal tumors. b Representative micrographs showing TCF1 (brown) and CD4 (red) immunopositivity in luminal A tumors and TNBC. Arrowheads indicate TCF1+ cells and double arrowheads indicate TCF1+/CD4+ cells (scale bar: 50μm). c Quantification of the assessment of TCF1 (%) expression by cancer cells, showing significantly decreased expression in TNBC versus luminal A tumors.Supplementary Figure 3. a-b Kaplan–Meier survival curve employing KM plotter demonstrate that increased CD8A and TCF7 mRNA is associated with improved disease-free survival in breast cancer. c-d Kaplan–Meier survival analysis of TCF1s (stromal cells expressing TCF1) and TCF1c(%) (percentage of TCF1 expression by cancer cells) on the whole population. Supplementary Figure 4. Kaplan–Meier survival analysis of CD8+Ki67+ / CD8+ (a) and CD8+TCF1+ / CD8+ (b) on whole cohort. TCF1s (stromal cells expressing TCF1) and TCF1c(%) (percentage of TCF1 expression by cancer cells) on luminal A tumors (c, d) and TNBC (e, f). Supplementary Figure 5. a Kaplan–Meier survival analysis of PD-L1 status [assessment of PD-L1+ immune cells in the tumor microenvironment per high power field (HPF, magnification 400x)] in the whole population. b Kaplan–Meier survival curve employing KM plotter demonstrate that increased CD163 mRNA predicts poor disease-free survival in breast cancer. Supplementary Figure 6. Kaplan–Meier survival analysis of PD-L1 sta [file 262_2024_3630_MOESM1_ESM.pptx]

## Slide 1
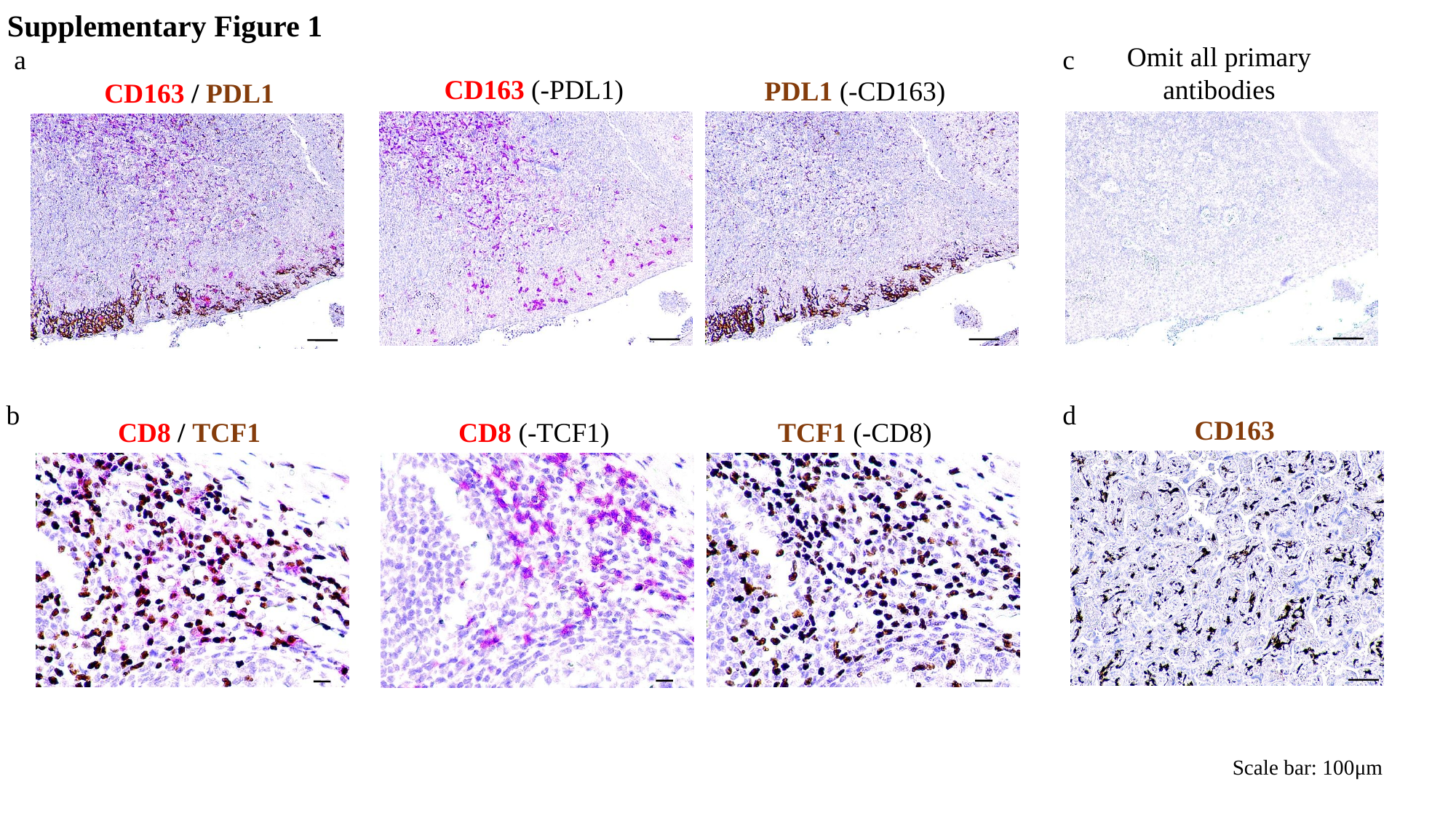

Supplementary Figure 1
Omit all primary antibodies
a
c
CD163 (-PDL1)
PDL1 (-CD163)
CD163 / PDL1
b
d
CD163
CD8 / TCF1
CD8 (-TCF1)
TCF1 (-CD8)
Scale bar: 100μm

## Slide 2
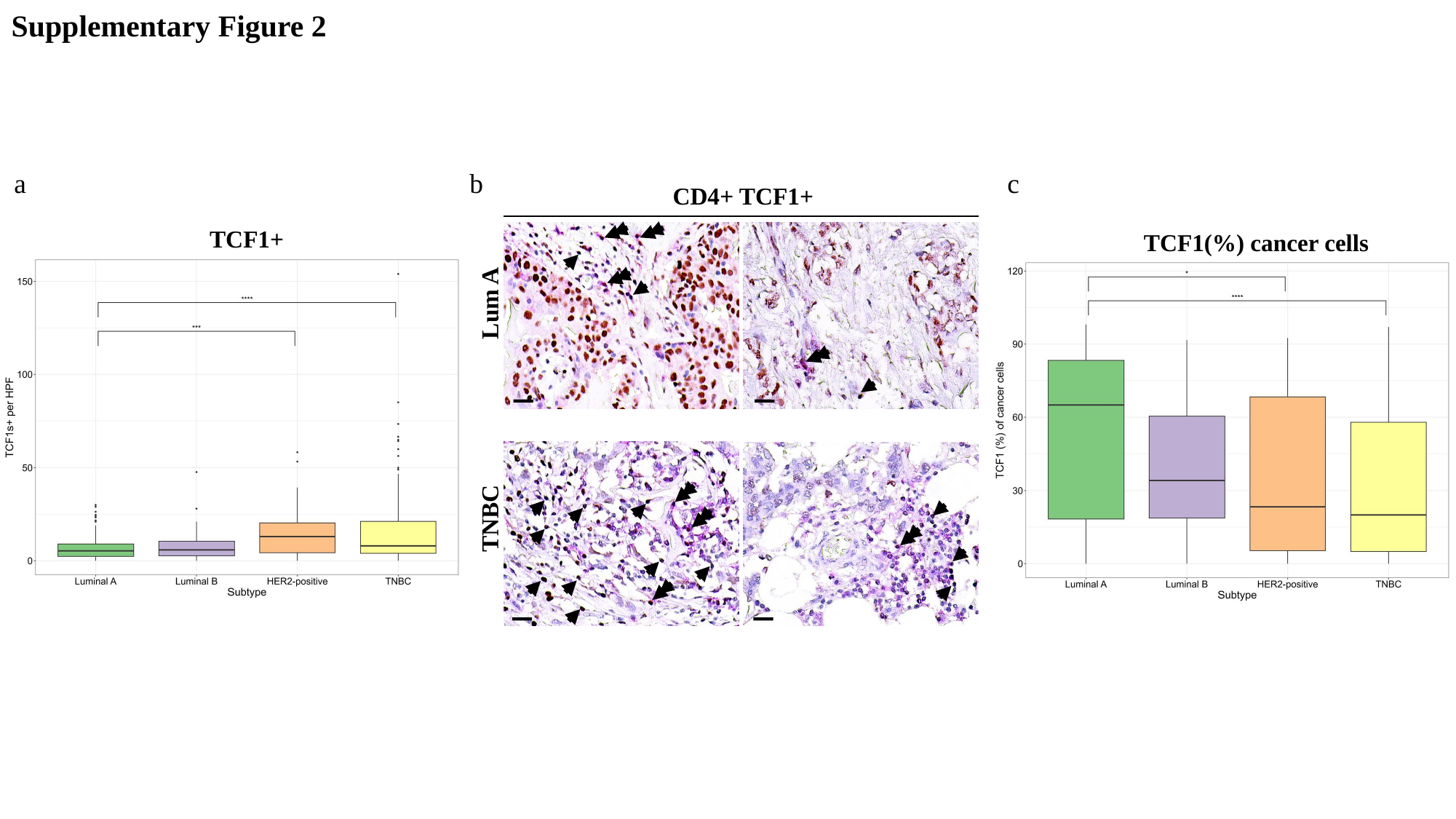

Supplementary Figure 2
a
b
c
CD4+ TCF1+
TCF1+
TCF1(%) cancer cells
Lum A
TNBC

## Slide 3
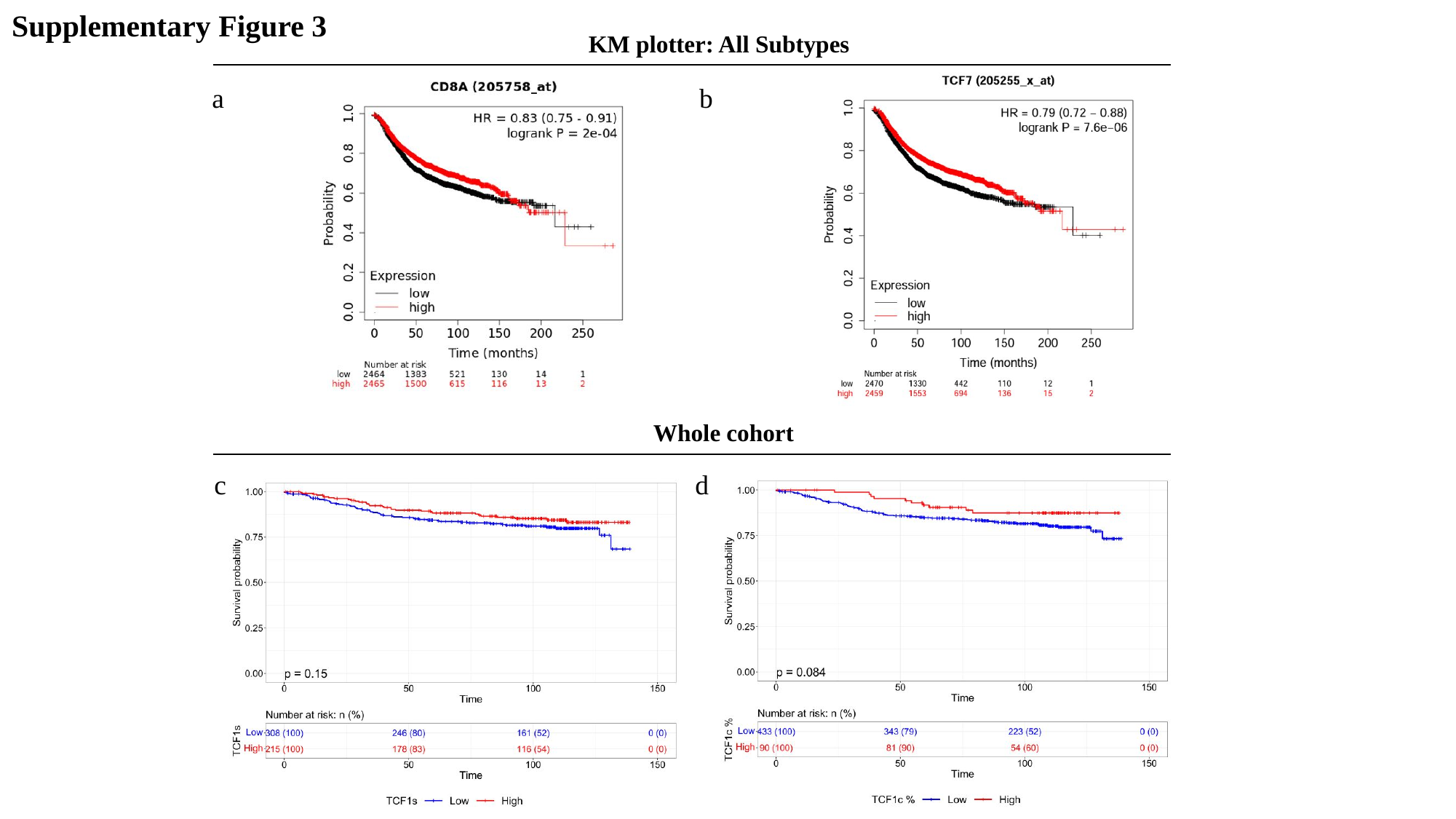

Supplementary Figure 3
KM plotter: All Subtypes
a
b
Whole cohort
c
d

## Slide 4
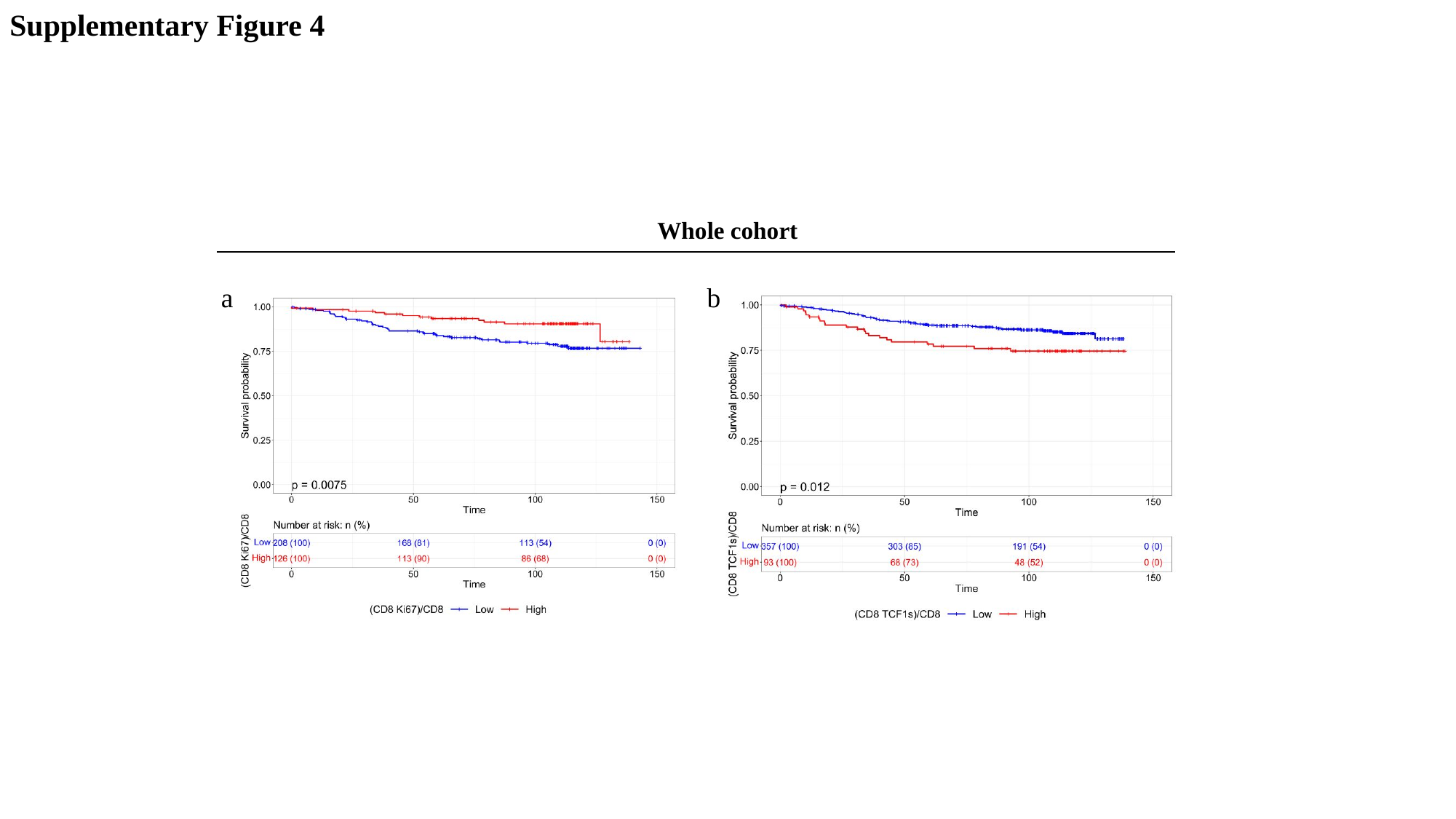

Supplementary Figure 4
Whole cohort
a
b

## Slide 5
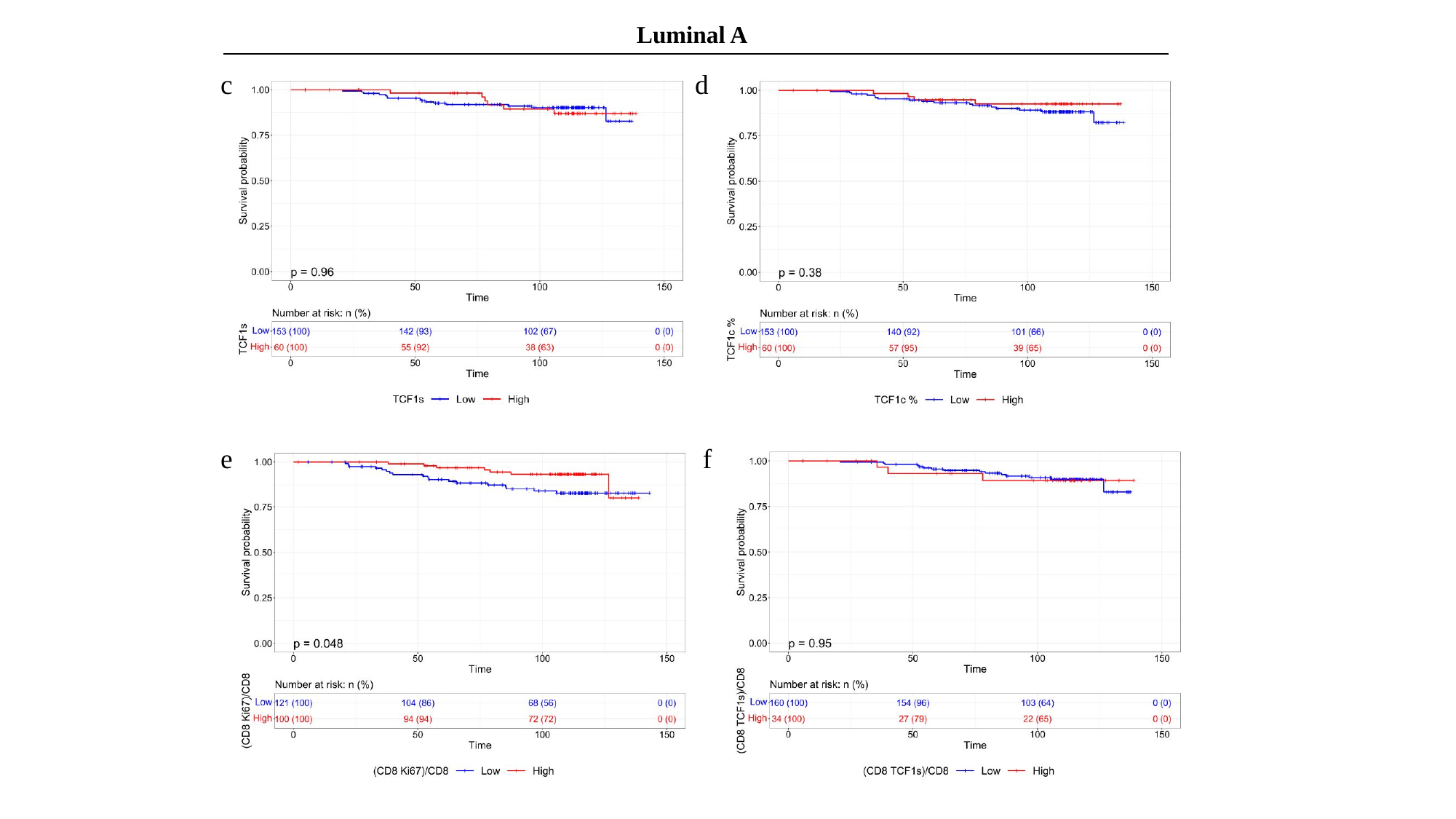

Luminal A
c
d
e
f

## Slide 6
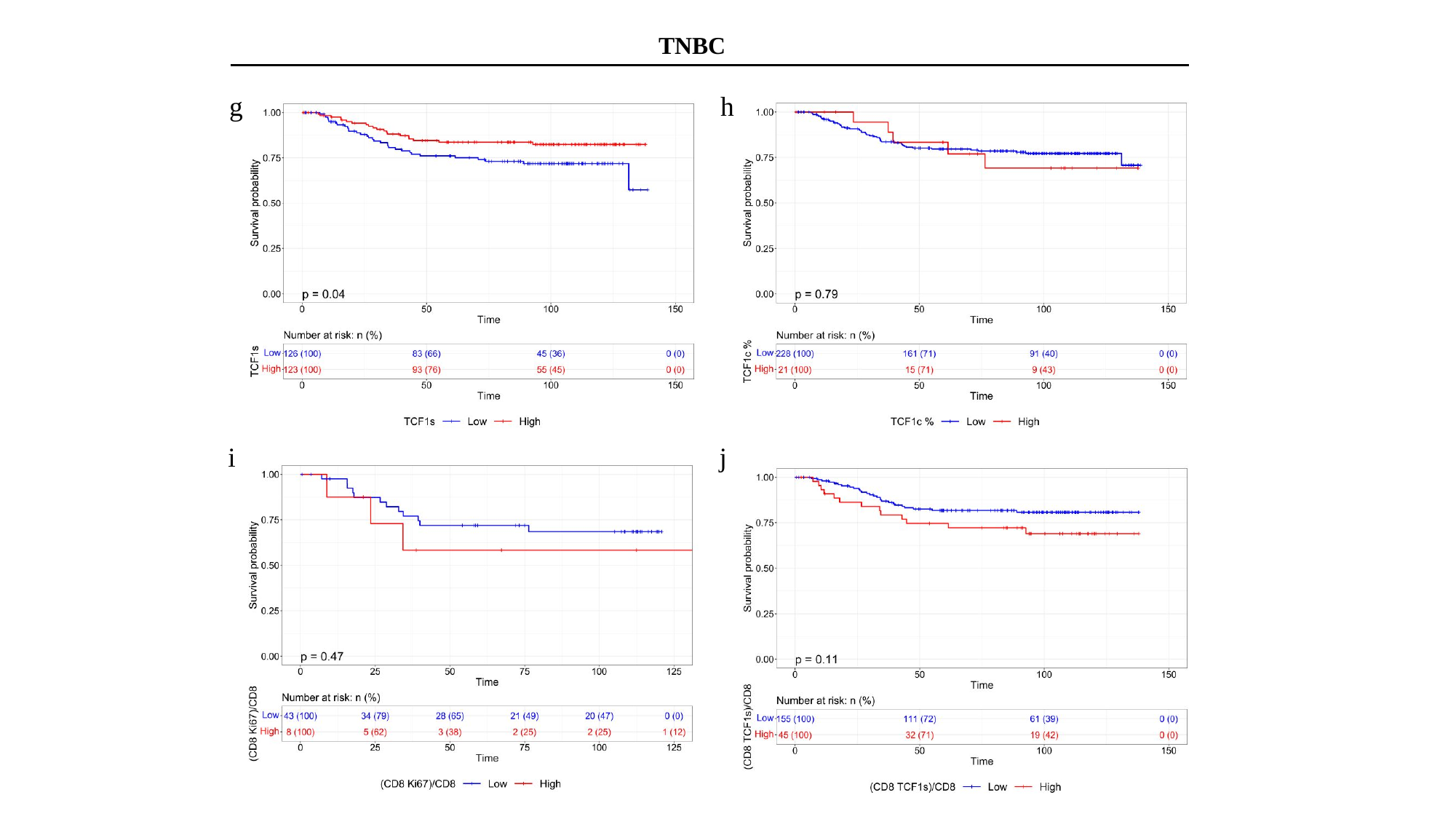

TNBC
g
h
i
j

## Slide 7
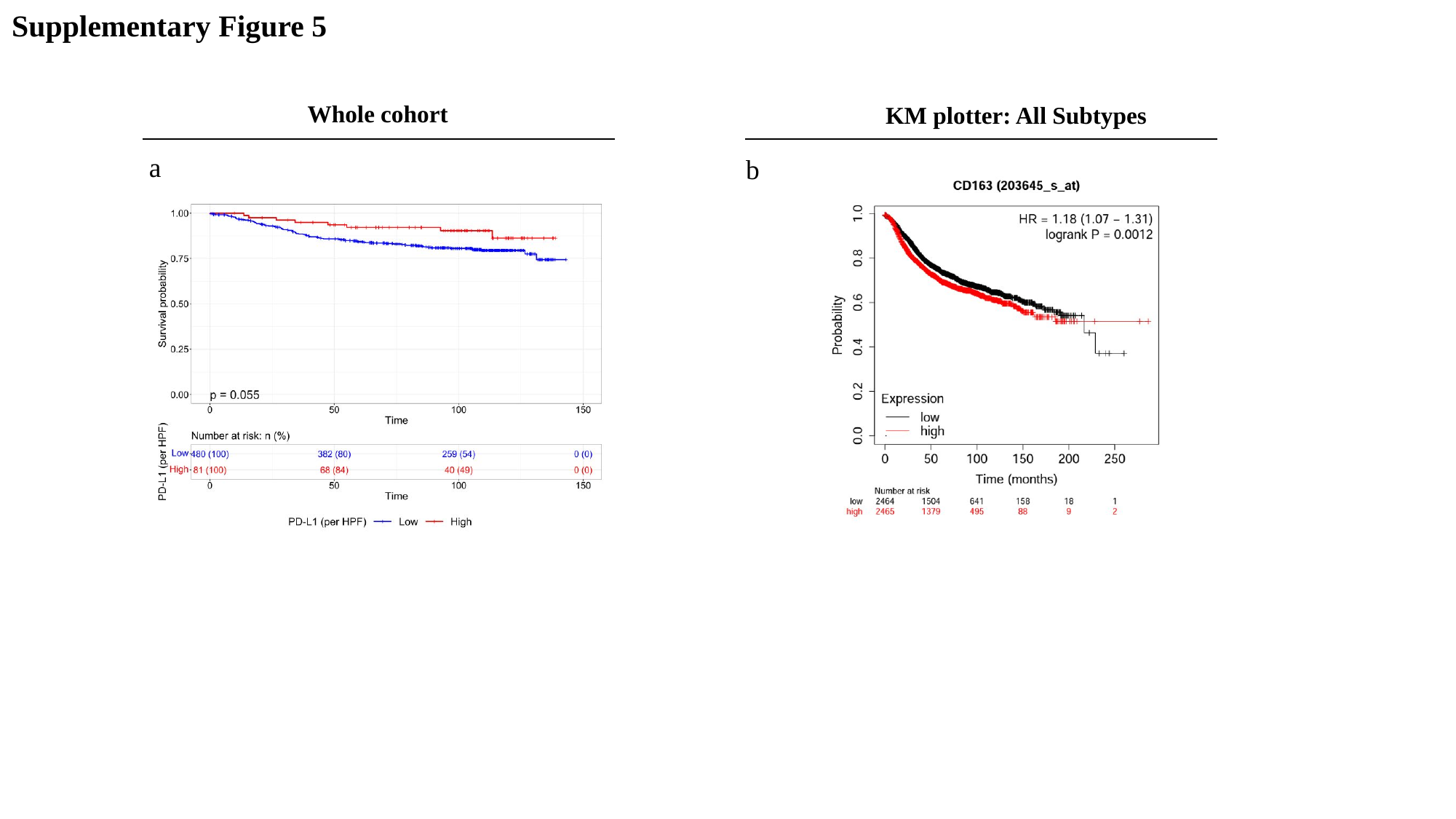

Supplementary Figure 5
Whole cohort
KM plotter: All Subtypes
a
b

## Slide 8
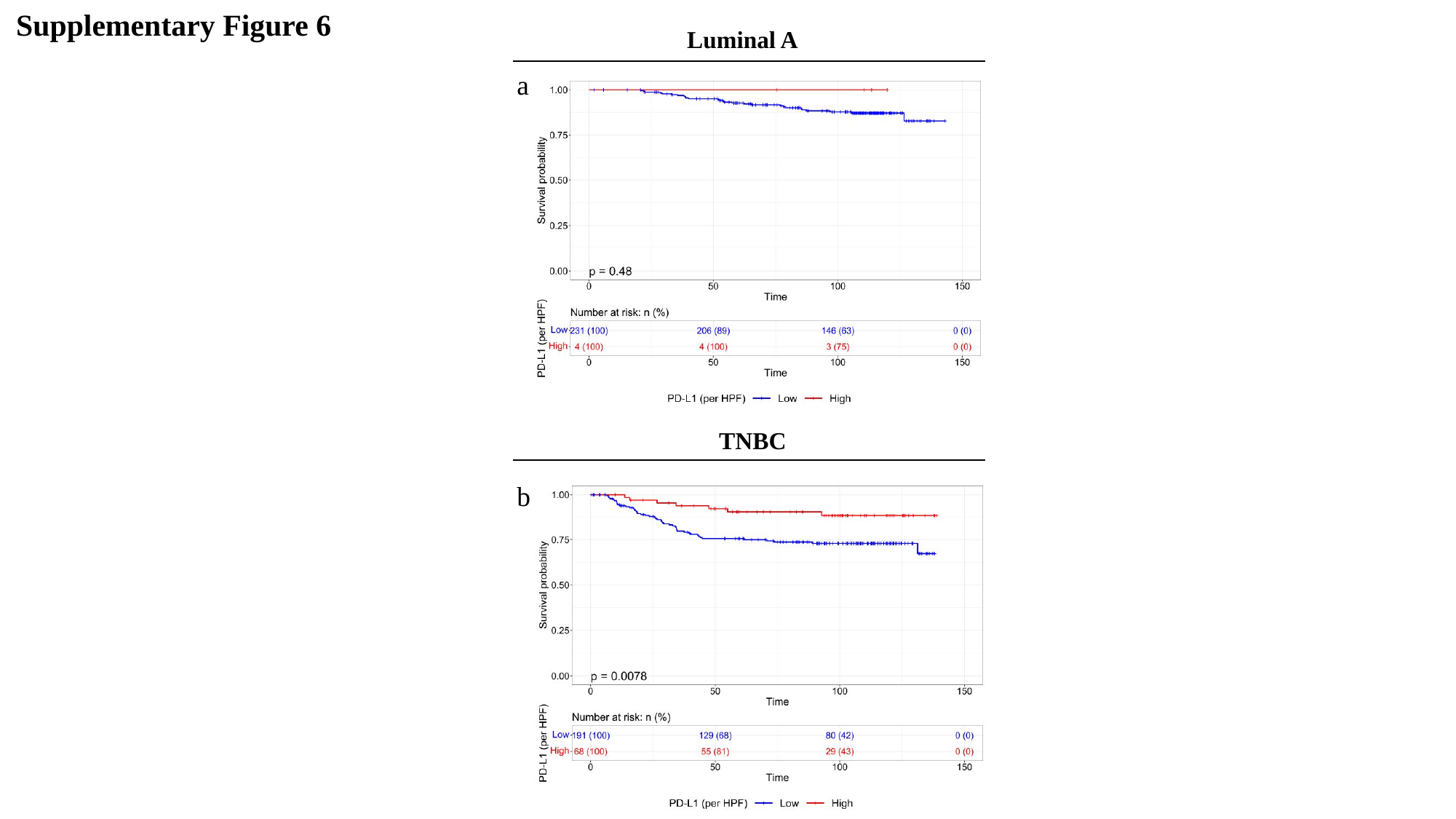

Supplementary Figure 6
Luminal A
a
TNBC
b

## Slide 9
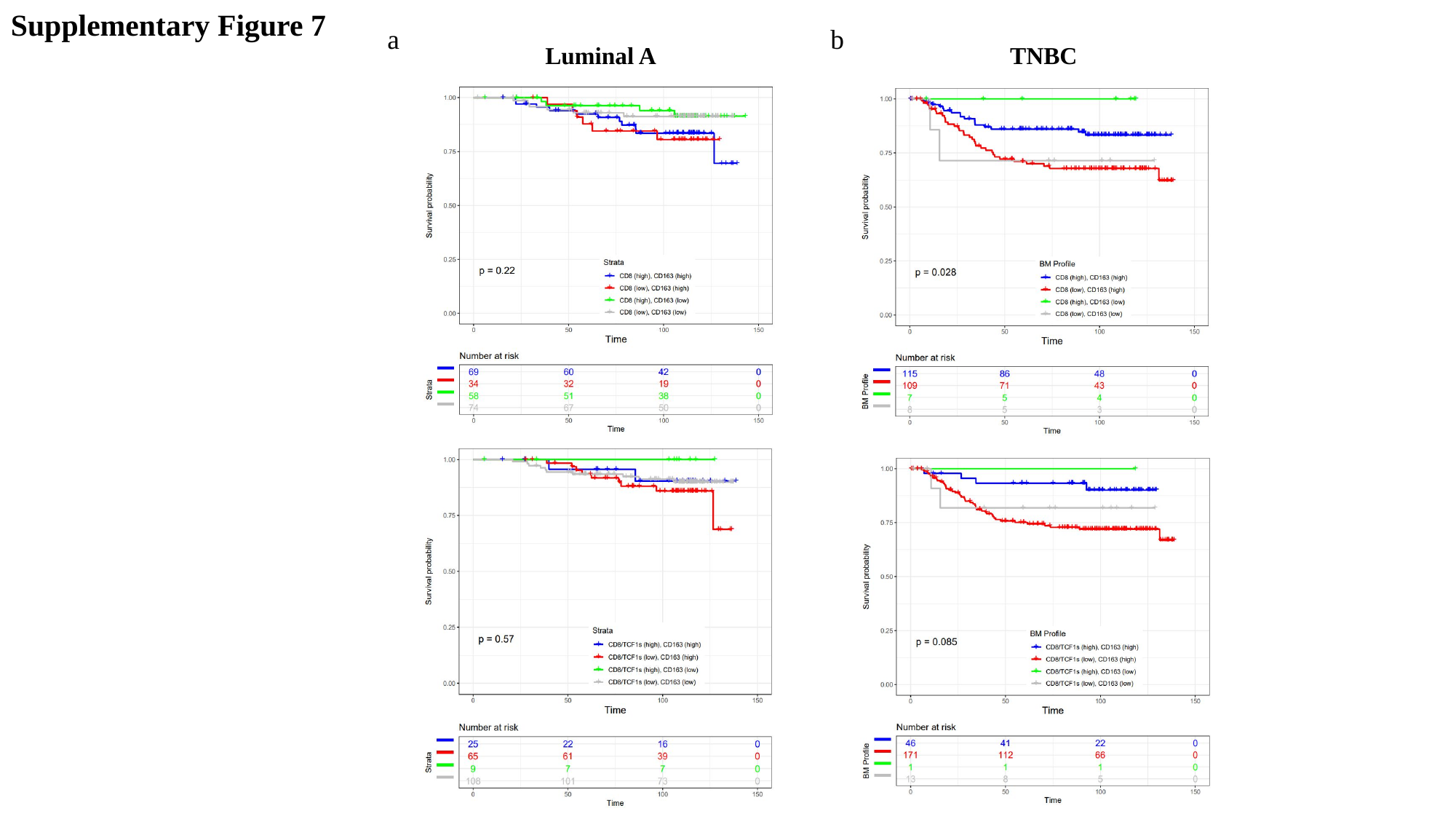

Supplementary Figure 7
a
b
Luminal A
TNBC

## Slide 10
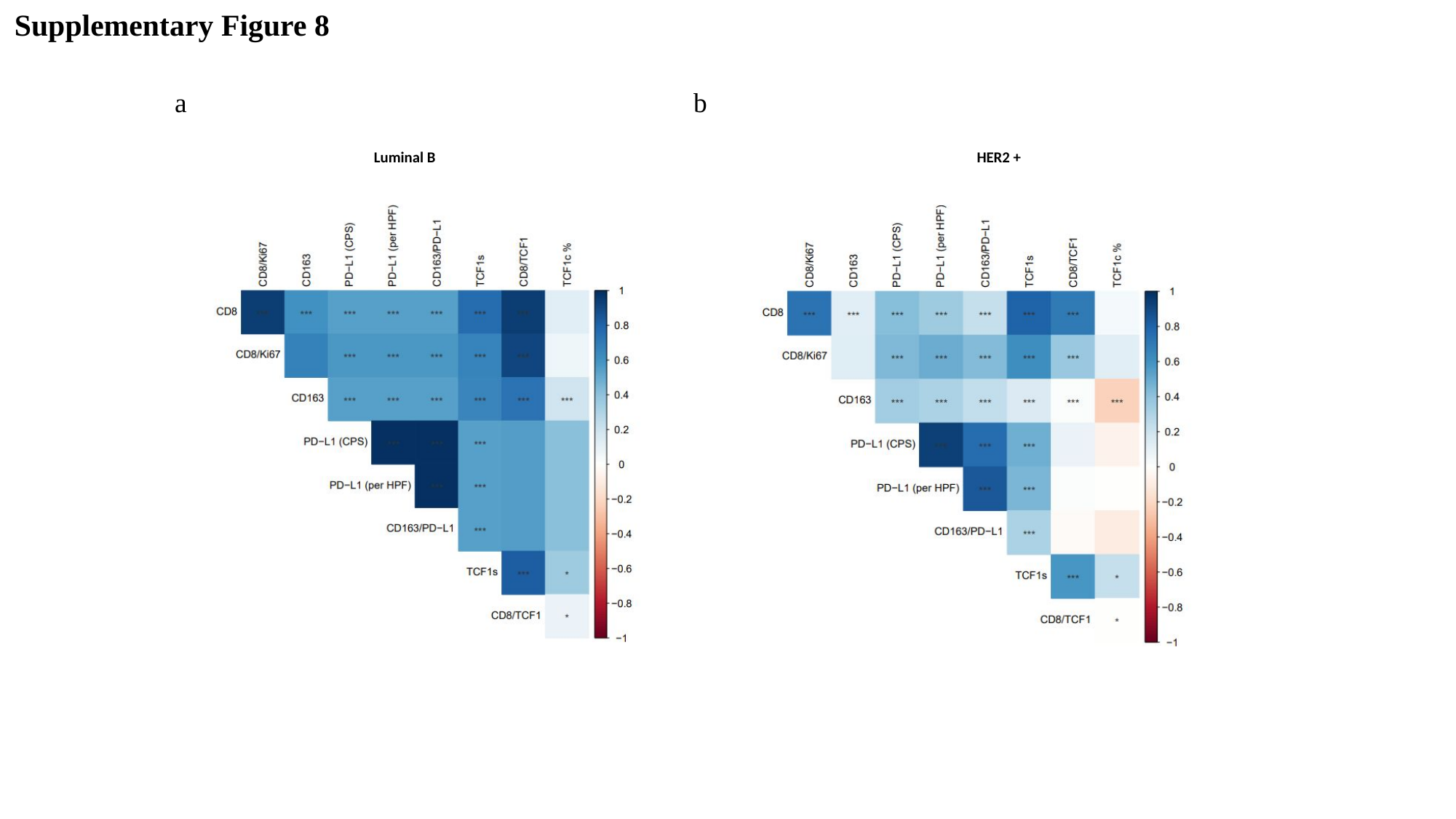

Supplementary Figure 8
a
b
Luminal B
HER2 +
