# Supplementary material for: Distinct profiles of proliferating CD8+/TCF1+ T cells and CD163+/PD-L1+ macrophages predict risk of relapse differently among treatment-naïve breast cancer subtypes
Source: Cancer Immunol Immunother. 2024 Feb 13;73(3):46. doi: 10.1007/s00262-024-03630-8 (PMC10864422; doi:10.1007/s00262-024-03630-8)
Supplement: Supplementary file 2 — Supplementary file2 (DOCX 48 kb) [file 262_2024_3630_MOESM2_ESM.docx]

**Supplementary Table 1.** Optimal cut-off values of the tested biomarkers, determined with the *cutp()* function of the *survMisc* package (R Foundation for statistical computing Vienna, Austria).

| **Biomarker** | **Cut-off** |
| --- | --- |
| CD8 | 2.57 |
| CD8/Ki-67 | 0.22 |
| CD163 | 2.333 |
| PD-L1 (CPS) | 1.149 |
| PD-L1 (per HPF) | 0.7 |
| CD163/PD-L1 | 0.25 |
| TCF1s | 8.333 |
| CD8/TCF1 | 1.667 |
| TCF1c % | 81.667 |
| (CD8+Ki67+)/CD8+ | 0.420 |
| (CD8+TCF1s+)/CD8+ | 0.300 |

**Supplementary Table 2**: Expression of the tested biomarkers among the breast cancer subtypes in the whole patient cohort.

| **Biomarker** | **Mean ± SD** | | | | **Median (Q1-Q3)** | | | | **P-value** |
| --- | --- | --- | --- | --- | --- | --- | --- | --- | --- |
|  | **Luminal A**  **(N=284)** | **Luminal B**  **(N=28)** | **HER2-positive**  **(N=46)** | **TNBC**  **(N=433)** | **Luminal A**  **(N=284)** | **Luminal B**  **(N=28)** | **HER2-positive**  **(N=46)** | **TNBC**  **(N=433)** |  |
| **CD8+ per HPF** | 5.056 ± 5.909 | 5.366 ± 8.349 | 10.73 ± 9.547 | 9.128 ± 15.27 | 2.850  (0.83 – 7.166) | 2.002  (0.33 – 6.150) | 7.092  (3.274 – 16.96) | 3.000  (0.33 – 11.17) | **0.002** |
| **CD8+ Ki67+ per HPF** | 1.784 ± 2.061 | 1.255 ± 1.606 | 2.173 ± 2.400 | 2.493 ± 2.779 | 1.085  (0.25 – 2.670) | 0.67  (0 – 2.500) | 1.500  (0.57 – 2.800) | 1.800  (0.14 – 3.800) | 0.135 |
| **CD163+ per HPF** | 4.507 ± 7.099 | 11.37 ± 11.72 | 12.75 ± 11.71 | 25.04 ± 21.79 | 1.708  (0.33 – 5.333) | 7.333  (2.833 – 16.67) | 10.67  (4.667 – 16.67) | 19.00  (8.667 – 37.50) | **<0.001** |
| **PD-L1+ (CPS)** | 0.089 ± 0.46 | 0.84 ± 2.618 | 2.297 ± 7.033 | 3.307 ± 8.885 | 0  (0 – 0) | 0  (0 – 0) | 0  (0 – 0) | 0  (0 – 1.548) | **<0.001** |
| **PD-L1+ per HPF** | 0.056 ± 0.35 | 0.77 ± 2.762 | 0.71 ± 2.529 | 2.497 ± 7.001 | 0  (0 – 0) | 0  (0 – 0) | 0  (0 – 0) | 0  (0 – 0.70) | **<0.001** |
| **CD163+ PD-L1+ per HPF** | 0.013 ± 0.12 | 0.43 ± 2.065 | 0.30 ± 1.330 | 0.68 ± 2.844 | 0  (0 – 0) | 0  (0 – 0) | 0  (0 – 0) | 0  (0 – 0) | **<0.001** |
| **TCF1s+ per HPF** | 6.510 ± 5.720 | 9.382 ± 10.95 | 16.61 ± 15.03 | 15.22 ± 17.68 | 5.333  (2.333 – 9.000) | 5.834  (2.667 – 11.00) | 13.00  (4.333 – 20.33) | 8.000  (4.000 – 21.33) | **<0.001** |
| **CD8+ TCF1s+ per HPF** | 0.89 ± 1.895 | 1.472 ± 2.929 | 1.904 ± 3.884 | 1.638 ± 6.050 | 0  (0 – 1.0) | 0.33  (0 – 1.166) | 1.0  (0 – 1.667) | 0  (0 – 1.0) | **0.017** |
| **TCF1 (%) of cancer cells** | 53.23 ± 34.33 | 40.20 ± 29.97 | 34.45 ± 32.69 | 30.67 ± 29.84 | 65.00  (18.33 – 83.33) | 34.17  (15.83 – 64.17) | 23.33  (5.333 – 68.33) | 20.00  (5.000 – 58.33) | **<0.001** |
| **(CD8+ Ki67+)/CD8+ ratio per HPF** | 0.48 ± 0.68 | 0.23 ± 0.23 | 0.26 ± 0.25 | 0.26 ± 0.23 | 0.38  (0.17 – 0.62) | 0.12  (0 – 0.40) | 0.18  (0.065 – 0.47) | 0.23  (0.058 – 0.38) | **<0.001** |
| **(CD8+ TCF1s+)/CD8+ ratio per HPF** | 0.14 ± 0.20 | 0.36 ± 0.45 | 0.17 ± 0.20 | 0.16 ± 0.22 | 0  (0 – 0.22) | 0.28  (0.036 – 0.48) | 0.12  (0 – 0.23) | 0.062  (0 – 0.25) | **0.006** |

Note: The Kruskal-Wallis one-way ANOVA test was used for the comparisons.

**Supplementary Table 3**: Expression of the tested biomarkers among the breast cancer subtypes in the Stage I/II patients.

| **Biomarker** | **Mean ± SD** | | | | **Median (Q1-Q3)** | | | | **P-value** |
| --- | --- | --- | --- | --- | --- | --- | --- | --- | --- |
|  | **Luminal A**  **(N=258)** | **Luminal B**  **(N=27)** | **HER2-positive**  **(N=38)** | **TNBC**  **(N=341)** | **Luminal A**  **(N=258)** | **Luminal B**  **(N=27)** | **HER2-positive**  **(N=38)** | **TNBC**  **(N=341)** |  |
| **CD8+ per HPF** | 5.012 ± 5.616 | 5.590 ± 8.452 | 11.50 ± 10.00 | 9.849 ± 16.23 | 2.930  (0.83 – 7.250) | 2.251  (0.42 – 6.360) | 6.934  (4.085 – 18.00) | 3.000  (0.50 – 11.67) | **0.003** |
| **CD8+ Ki67+ per HPF** | 1.832 ± 2.123 | 1.307 ± 1.618 | 2.016 ± 2.237 | 2.678 ± 2.873 | 1.170  (0.22 – 2.670) | 0.67  (0 – 2.500) | 1.500  (0.50 – 2.800) | 2.000  (0.22 – 3.735) | 0.188 |
| **CD163+ per HPF** | 4.222 ± 6.569 | 11.15 ± 11.92 | 13.73 ± 12.58 | 26.39 ± 22.13 | 2.000  (0.33 – 5.166) | 7.000  (2.250 – 16.50) | 11.33  (4.667 – 17.33) | 20.33  (9.500 – 39.30) | **<0.001** |
| **PD-L1+ (CPS)** | 0.098 ± 0.48 | 0.88 ± 2.668 | 2.510 ± 7.640 | 4.126 ± 9.931 | 0  (0 – 0) | 0  (0 – 0) | 0  (0 – 0.37) | 0  (0 – 3.700) | **<0.001** |
| **PD-L1+ per HPF** | 0.061 ± 0.37 | 0.80 ± 2.817 | 0.78 ± 2.772 | 3.047 ± 7.815 | 0  (0 – 0) | 0  (0 – 0) | 0  (0 – 0) | 0  (0 – 1.667) | **<0.001** |
| **CD163+ PD-L1+ per HPF** | 0.014 ± 0.13 | 0.44 ± 2.107 | 0.36 ± 1.474 | 0.82 ± 3.195 | 0  (0 – 0) | 0  (0 – 0) | 0  (0 – 0) | 0  (0 – 0) | **<0.001** |
| **TCF1s+ per HPF** | 6.540 ± 5.615 | 9.790 ± 11.01 | 17.71 ± 15.70 | 15.93 ± 18.71 | 5.333  (2.333 – 9.000) | 6.000  (2.667 – 12.00) | 15.67  (4.333 – 21.33) | 8.000  (4.333 – 22.67) | **<0.001** |
| **CD8+ TCF1s+ per HPF** | 0.89 ± 1.854 | 1.536 ± 2.978 | 2.025 ± 4.216 | 1.808 ± 6.665 | 0  (0 – 1.0) | 0.33  (0 – 1.333) | 1.0  (0 – 1.667) | 0.33  (0 – 1.333) | **0.016** |
| **TCF1 (%) of cancer cells** | 53.86 ± 33.80 | 41.51 ± 29.93 | 32.61 ± 33.11 | 31.10 ± 29.56 | 65.00  (21.83 – 83.83) | 35.00  (21.67 – 71.67) | 16.67  (5.000 – 68.33) | 20.00  (6.667 – 56.67) | **<0.001** |
| **(CD8+ Ki67+)/CD8+ ratio per HPF** | 0.48 ± 0.71 | 0.23 ± 0.23 | 0.21 ± 0.21 | 0.24 ± 0.20 | 0.38  (0.19 – 0.62) | 0.12  (0 – 0.40) | 0.11  (0.052 – 0.37) | 0.23  (0.068 – 0.34) | **<0.001** |
| **(CD8+ TCF1s+)/CD8+ ratio per HPF** | 0.14 ± 0.20 | 0.36 ± 0.45 | 0.16 ± 0.19 | 0.15 ± 0.22 | 0  (0 – 0.23) | 0.28  (0.036 – 0.48) | 0.12  (0 – 0.22) | 0.062  (0 – 0.20) | **0.008** |

Note: The Kruskal-Wallis one-way ANOVA test was used for the comparisons.

**Supplementary Table 4**: Expression of the tested biomarkers among the breast cancer subtypes in the Stage III/IV patients.

| **Biomarker** | **Mean ± SD** | | | **Median (Q1-Q3)** | | | **P-value** |
| --- | --- | --- | --- | --- | --- | --- | --- |
|  | **Luminal A**  **(N=24)** | **HER2-positive**  **(N=7)** | **TNBC**  **(N=82)** | **Luminal A**  **(N=24)** | **HER2-positive**  **(N=7)** | **TNBC**  **(N=82)** |  |
| **CD8+ per HPF** | 5.631 ± 8.474 | 7.075 ± 7.324 | 6.678 ± 10.98 | 2.416  (0.75 – 6.166) | 4.875  (1.548 – 10.65) | 1.500  (0.33 – 6.548) | 0.430 |
| **CD8+ Ki67+ per HPF** | 1.320 ± 1.301 | 2.902 ± 3.410 | 2.432 ± 2.730 | 0.88  (0.33 – 2.170) | 1.300  (0.67 – 4.140) | 1.330  (0.14 – 3.875) | 0.441 |
| **CD163+ per HPF** | 7.524 ± 11.08 | 8.222 ± 6.145 | 21.79 ± 20.53 | 1.333  (0 – 12.33) | 6.500  (4.667 – 15.00) | 14.67  (8.300 – 28.33) | **<0.001** |
| **PD-L1+ (CPS)** | 0 ± 0 | 1.616 ± 3.958 | 0.61 ± 1.977 | 0  (0 – 0) | 0  (0 – 0) | 0  (0 – 0) | 0.073 |
| **PD-L1+ per HPF** | 0 ± 0 | 0.47 ± 1.143 | 0.71 ± 2.210 | 0  (0 – 0) | 0  (0 – 0) | 0  (0 – 0) | 0.071 |
| **CD163+ PD-L1+ per HPF** | 0 ± 0 | 0.067 ± 0.16 | 0.22 ± 0.89 | 0  (0 – 0) | 0  (0 – 0) | 0  (0 – 0) | 0.276 |
| **TCF1s+ per HPF** | 6.230 ± 6.766 | 11.64 ± 11.34 | 12.85 ± 13.23 | 3.667  (1.333 – 9.667) | 7.750  (3.333 – 19.00) | 8.666  (3.500 – 18.50) | 0.118 |
| **CD8+ TCF1s+ per HPF** | 0.87 ± 2.299 | 1.361 ± 1.916 | 1.045 ± 2.856 | 0  (0 – 1.0) | 0.33  (0 – 3.000) | 0  (0 – 1.0) | 0.614 |
| **TCF1 (%) of cancer cells** | 47.44 ± 39.44 | 42.72 ± 32.25 | 29.04 ± 31.52 | 56.67  (5.000 – 80.00) | 36.50  (23.33 – 71.67) | 16.67  (0 – 60.00) | 0.112 |
| **(CD8+ Ki67+)/CD8+ ratio per HPF** | 0.42 ± 0.39 | 0.52 ± 0.30 | 0.29 ± 0.28 | 0.34  (0.15 – 0.64) | 0.52  (0.27 – 0.65) | 0.27  (0.026 – 0.41) | 0.255 |
| **(CD8+ TCF1s+)/CD8+ ratio per HPF** | 0.11 ± 0.15 | 0.19 ± 0.24 | 0.18 ± 0.24 | 0.038  (0 – 0.17) | 0.12  (0 – 0.28) | 0.054  (0 – 0.33) | 0.599 |

Notes: The Kruskal-Wallis one-way ANOVA test was used for the comparisons. As we only had one Luminal type B Stage III/IV breast cancer case, this was excluded from this particular analysis.

**Supplementary Table 5**: Comparison of the biomarkers’ expression between the Stage I/II and Stage III/IV breast cancer patients in the whole cohort.

| **Biomarker** | **Mean ± SD** | | **Median (Q1-Q3)** | | **P-value** |
| --- | --- | --- | --- | --- | --- |
|  | **Stages I/II**  **(N=664)** | **Stages III/IV**  **(N=114)** | **Stages I/II**  **(N=664)** | **Stages III/IV**  **(N=114)** |  |
| **CD8+ per HPF** | 7.490 ± 11.88 | 6.340 ± 10.00 | 3.165  0.67 – 8.916 | 2.251  0.50 – 6.899 | 0.122 |
| **CD8+ Ki67+ per HPF** | 1.897 ± 2.200 | 1.950 ± 2.328 | 1.300  0.17 – 2.700 | 1.085  0.25 – 3.000 | 0.983 |
| **CD163+ per HPF** | 14.64 ± 18.83 | 17.13 ± 18.81 | 6.300  1.667 – 20.00 | 11.14  3.333 – 21.30 | 0.061 |
| **PD-L1+ (CPS)** | 2.010 ± 7.050 | 0.52 ± 1.923 | 0  0 – 0 | 0  0 – 0 | 0.192 |
| **PD-L1+ per HPF** | 1.419 ± 5.380 | 0.51 ± 1.842 | 0  0 – 0 | 0  0 – 0 | 0.380 |
| **CD163+ PD-L1+ per HPF** | 0.40 ± 2.202 | 0.15 ± 0.73 | 0  0 – 0 | 0  0 – 0 | 0.209 |
| **TCF1s+ per HPF** | 11.56 ± 14.56 | 10.86 ± 11.94 | 7.000  3.333 – 14.50 | 6.834  2.667 – 13.00 | 0.628 |
| **CD8+ TCF1s+ per HPF** | 1.402 ± 4.792 | 1.010 ± 2.620 | 0.17  0 – 1.0 | 0  0 – 1.0 | 0.331 |
| **TCF1 (%) of cancer cells** | 41.80 ± 33.48 | 34.66 ± 34.32 | 33.33  11.67 – 75.00 | 19.50  1.667 – 71.67 | **0.024** |
| **(CD8+ Ki67+)/CD8+ ratio per HPF** | 0.41 ± 0.62 | 0.38 ± 0.34 | 0.33  0.11 – 0.55 | 0.34  0.12 – 0.57 | 0.897 |
| **(CD8+ TCF1s+)/CD8+ ratio per HPF** | 0.16 ± 0.23 | 0.16 ± 0.21 | 0.065  0 – 0.25 | 0.054  0 – 0.31 | 0.984 |

Notes: The Mann-Whitney’s U test was used for the comparisons. Information regarding tumor stage was available for 778/791 breast cancer cases evaluated in this study.

**Supplementary Table 6**: Comparison of the biomarkers’ expression between the Stage I/II and Stage III/IV Luminal type A breast cancer patients.

| **Biomarker** | **Mean ± SD** | | **Median (Q1-Q3)** | | **P-value** |
| --- | --- | --- | --- | --- | --- |
|  | **Stages I/II**  **(N=258)** | **Stages III/IV**  **(N=24)** | **Stages I/II**  **(N=258)** | **Stages III/IV**  **(N=24)** |  |
| **CD8+ per HPF** | 5.012 ± 5.616 | 5.631 ± 8.474 | 2.930  (0.83 – 7.250) | 2.416  (0.75 – 6.166) | 0.811 |
| **CD8+ Ki67+ per HPF** | 1.832 ± 2.123 | 1.320 ± 1.301 | 1.170  (0.22 – 2.670) | 0.88  (0.33 – 2.170) | 0.585 |
| **CD163+ per HPF** | 4.222 ± 6.569 | 7.524 ± 11.08 | 2.000  (0.33 – 5.166) | 1.333  (0 – 12.33) | 0.980 |
| **PD-L1+ (CPS)** | 0.098 ± 0.48 | 0 ± 0 | 0  (0 – 0) | 0  (0 – 0) | 0.198 |
| **PD-L1+ per HPF** | 0.061 ± 0.37 | 0 ± 0 | 0  (0 – 0) | 0  (0 – 0) | 0.213 |
| **CD163+ PD-L1+ per HPF** | 0.014 ± 0.13 | 0 ± 0 | 0  (0 – 0) | 0  (0 – 0) | 0.530 |
| **TCF1s+ per HPF** | 6.540 ± 5.615 | 6.230 ± 6.766 | 5.333  (2.333 – 9.000) | 3.667  (1.333 – 9.667) | 0.530 |
| **CD8+ TCF1s+ per HPF** | 0.89 ± 1.854 | 0.87 ± 2.299 | 0  (0 – 1.0) | 0  (0 – 1.0) | 0.977 |
| **TCF1 (%) of cancer cells** | 53.86 ± 33.80 | 47.44 ± 39.44 | 65.00  (21.83 – 83.83) | 56.67  (5.000 – 80.00) | 0.388 |
| **(CD8+ Ki67+)/CD8+ ratio per HPF** | 0.48 ± 0.71 | 0.42 ± 0.39 | 0.38  (0.19 – 0.62) | 0.34  (0.15 – 0.64) | 0.871 |
| **(CD8+ TCF1s+)/CD8+ ratio per HPF** | 0.14 ± 0.20 | 0.11 ± 0.15 | 0  (0 – 0.23) | 0.038  (0 – 0.17) | 0.740 |

Note: The Mann-Whitney’s U test was used for the comparisons.

**Supplementary Table 7**: Comparison of the biomarkers’ expression between the Stage I/II and Stage III/IV TNBC patients.

| **Biomarker** | **Mean ± SD** | | **Median (Q1-Q3)** |  | **P-value** |
| --- | --- | --- | --- | --- | --- |
|  | **Stages I/II**  **(N=341)** | **Stages III/IV**  **(N=82)** | **Stages I/II**  **(N=341)** | **Stages III/IV**  **(N=82)** |  |
| **CD8+ per HPF** | 9.849 ± 16.23 | 6.678 ± 10.98 | 3.000  (0.50 – 11.67) | 1.500  (0.33 – 6.548) | 0.167 |
| **CD8+ Ki67+ per HPF** | 2.678 ± 2.873 | 2.432 ± 2.730 | 2.000  (0.22 – 3.735) | 1.330  (0.14 – 3.875) | 0.728 |
| **CD163+ per HPF** | 26.39 ± 22.13 | 21.79 ± 20.53 | 20.33  (9.500 – 39.30) | 14.67  (8.300 – 28.33) | 0.124 |
| **PD-L1+ (CPS)** | 4.126 ± 9.931 | 0.61 ± 1.977 | 0  (0 – 3.700) | 0  (0 – 0) | **0.015** |
| **PD-L1+ per HPF** | 3.047 ± 7.815 | 0.71 ± 2.210 | 0  (0 – 1.667) | 0  (0 – 0) | 0.067 |
| **CD163+ PD-L1+ per HPF** | 0.82 ± 3.195 | 0.22 ± 0.89 | 0  (0 – 0) | 0  (0 – 0) | **0.028** |
| **TCF1s+ per HPF** | 15.93 ± 18.71 | 12.85 ± 13.23 | 8.000  (4.333 – 22.67) | 8.666  (3.500 – 18.50) | 0.299 |
| **CD8+ TCF1s+ per HPF** | 1.808 ± 6.665 | 1.045 ± 2.856 | 0.33  (0 – 1.333) | 0  (0 – 1.0) | 0.279 |
| **TCF1 (%) of cancer cells** | 31.10 ± 29.56 | 29.04 ± 31.52 | 20.00  (6.667 – 56.67) | 16.67  (0 – 60.00) | 0.269 |
| **(CD8+ Ki67+)/CD8+ ratio per HPF** | 0.24 ± 0.20 | 0.29 ± 0.28 | 0.23  (0.068 – 0.34) | 0.27  (0.026 – 0.41) | 0.722 |
| **(CD8+ TCF1s+)/CD8+ ratio per HPF** | 0.15 ± 0.22 | 0.18 ± 0.24 | 0.062  (0 – 0.20) | 0.054  (0 – 0.33) | 0.842 |

Note: The Mann-Whitney’s U test was used for the comparisons.

**Supplementary Table 8**: Univariate Cox regression analysis for DFS in the Luminal type A breast cancer patients, evaluating the prognostic impact of the biomarkers tested.

| **Biomarker** | **HR** | **95% CI** | **P-value** |
| --- | --- | --- | --- |
| CD8+ per HPF | 1.025 | 0.492-2.134 | 0.947 |
| CD8+ Ki67+ per HPF | 0.702 | 0.319-1.542 | 0.378 |
| CD163+ per HPF | 2.246 | 1.028-4.909 | **0.042** |
| TCF1s+ per HPF | 1.025 | 0.397-2.645 | 0.959 |
| CD8+ TCF1s+ per HPF | 0.598 | 0.139-2.569 | 0.49 |
| TCF1 (%) of cancer cells | 0.616 | 0.207-1.832 | 0.384 |
| (CD8+ Ki67+)/CD8+ ratio per HPF | 0.425 | 0.177-1.018 | 0.055 |
| (CD8+ TCF1s+)/CD8+ ratio per HPF | 1.038 | 0.300-3.587 | 0.953 |

Note: The analysis is not shown for the biomarkers “PD-L1+ (CPS)”, “PD-L1+ per HPF”, and “CD163+ PD-L1+ per HPF”, as almost all Luminal type A cases showed low expression (expression below the cut-offs applied for this study; Supplementary Table 1), leading to a HR=0.00 and a 95% CI 0.00-Inf.

Abbreviations: HR, hazard ratio; DFS, disease-free survival; CPS, combined positive score; HPF, high power field

**Supplementary Table 9**: Multivariate Cox regression analysis for DFS in the Luminal type A breast cancer patients, evaluating the prognostic impact of each biomarker (adjusted for tumor stage).

| **Biomarker** | **Variable** | **HR** | **95% CI** | **P-value** |
| --- | --- | --- | --- | --- |
| **CD8+ per HPF** | CD8+ per HPF | 1.090 | 0.521-2.280 | 0.82 |
|  | Stage (III/IV vs. I/II) | 2.615 | 0.986-6.934 | 0.053 |
| **CD8+ Ki67+ per HPF** | CD8+ Ki67+ per HPF | 0.692 | 0.315-1.522 | 0.36 |
|  | Stage (III/IV vs. I/II) | 2.534 | 0.962-6.674 | 0.06 |
| **CD163+ per HPF** | CD163+ per HPF | 2.360 | 1.077-5.170 | **0.032** |
|  | Stage (III/IV vs. I/II) | 2.967 | 1.113-7.906 | 0.03 |
| **TCF1s+ per HPF** | TCF1s+ per HPF | 1.048 | 0.406-2.706 | 0.923 |
|  | Stage (III/IV vs. I/II) | 2.907 | 0.969-8.724 | 0.057 |
| **CD8+ TCF1s+ per HPF** | CD8+ TCF1s+ per HPF | 0.674 | 0.155-2.931 | 0.599 |
|  | Stage (III/IV vs. I/II) | 2.756 | 0.911-8.337 | 0.073 |
| **TCF1 (%) of cancer cells** | TCF1 (%) per HPF | 0.652 | 0.218-1.944 | 0.443 |
|  | Stage (III/IV vs. I/II) | 2.791 | 0.928-8.393 | 0.068 |
| **(CD8+ Ki67+)/CD8+ ratio per HPF** | (CD8+ Ki67+)/CD8+ ratio per HPF | 0.405 | 0.169-0.974 | **0.043** |
|  | Stage (III/IV vs. I/II) | 3.303 | 1.226-8.897 | 0.018 |

Notes: For the Luminal type A subtype, the number of events was small to run multivariate Cox regression models with four variables, thus only two variables (each biomarker; tumor stage) were used as covariates in each model. In addition, the analysis is not shown for the biomarkers “PD-L1+ (CPS)”, “PD-L1+ per HPF”, and “CD163+ PD-L1+ per HPF”, as almost all Luminal type A cases showed low expression (according to the cut-offs used for this study; Supplementary Table 1), leading to a HR=0.00 and a 95% CI 0.00-Inf. For the biomarker “(CD8+ TCF1s+)/CD8+ ratio per HPF”, the number of events was small to run a multivariable Cox regression model, even with two variables.

Abbreviations: HR, hazard ratio; DFS, disease-free survival; CPS, combined positive score; HPF, high power field

**Supplementary Table 10**: Univariate Cox regression analysis for DFS in the TNBC patients, evaluating the prognostic impact of the biomarkers tested.

| **Biomarker** | **HR** | **95% CI** | **P-value** |
| --- | --- | --- | --- |
| CD8+ per HPF | 0.478 | 0.276-0.830 | **0.0087** |
| CD8+ Ki67+ per HPF | 0.652 | 0.237-1.794 | 0.407 |
| CD163+ per HPF | 1.217 | 0.380-3.898 | 0.741 |
| PD-L1+ (CPS) | 0.327 | 0.148-0.723 | **0.0058** |
| PD-L1+ per HPF | 0.357 | 0.161-0.789 | **0.011** |
| CD163+ PD-L1+ per HPF | 0.279 | 0.101-0.773 | **0.014** |
| TCF1s+ per HPF | 0.562 | 0.321-0.983 | **0.043** |
| CD8+ TCF1s+ per HPF | 0.268 | 0.097-0.745 | **0.012** |
| TCF1 (%) of cancer cells | 1.134 | 0.451-2.849 | 0.79 |
| (CD8+ Ki67+)/CD8+ ratio per HPF | 1.587 | 0.445-5.657 | 0.476 |
| (CD8+ TCF1s+)/CD8+ ratio per HPF | 1.709 | 0.882-3.312 | 0.113 |

Abbreviations: HR, hazard ratio; DFS, disease-free survival; CPS, combined positive score; HPF, high power field
